# Tracking the first electron transfer step at the donor side of oxygen-evolving photosystem II by time-resolved infrared spectroscopy

**DOI:** 10.1007/s11120-023-01057-3

**Published:** 2023-11-23

**Authors:** Mohamad Yahia Dekmak, Sarah M. Mäusle, Janosch Brandhorst, Philipp S. Simon, Holger Dau

**Affiliations:** 1 Department of Physics, Freie Universität Berlin, Berlin, Germany; 2 Molecular Biophysics and Integrated Bioimaging Division, Lawrence Berkeley National Laboratory, Berkeley, CA USA

**Keywords:** Chlorophyll donor, Oxygen evolution, Photosynthesis, Redox-active tyrosine, System response function, Water oxidation

## Abstract

In oxygen-evolving photosystem II (PSII), the multi-phasic electron transfer from a redox-active tyrosine residue (TyrZ) to a chlorophyll cation radical (P680^+^) precedes the water-oxidation chemistry of the S-state cycle of the Mn_4_Ca cluster. Here we investigate these early events, observable within about 10 ns to 10 ms after laser-flash excitation, by time-resolved single-frequency infrared (IR) spectroscopy in the spectral range of 1310–1890 cm^−1^ for oxygen-evolving PSII membrane particles from spinach. Comparing the IR difference spectra at 80 ns, 500 ns, and 10 µs allowed for the identification of quinone, P680 and TyrZ contributions. A broad electronic absorption band assignable P680^+^ was used to trace largely specifically the P680^+^ reduction kinetics. The experimental time resolution was taken into account in least-square fits of P680^+^ transients with a sum of four exponentials, revealing two nanosecond phases (30–46 ns and 690–1110 ns) and two microsecond phases (4.5–8.3 µs and 42 µs), which mostly exhibit a clear S-state dependence, in agreement with results obtained by other methods. Our investigation paves the road for further insight in the early events associated with TyrZ oxidation and their role in the preparing the PSII donor side for the subsequent water oxidation chemistry.

**Supplementary Information:**

The online version contains supplementary material available at 10.1007/s11120-023-01057-3.

## Introduction

Photosynthesis is a fundamentally important process to sustain aerobic life on planet Earth by (a) supplying organisms with chemical energy harvested from light energy and by (b) producing the oxygen we breathe. Artificial photosynthesis could contribute to mitigation of global climate change by sustainable production of non-fossil fuels, which is one of many reasons why studying the natural system in plants, cyanobacteria and algae is of interest to society (Dau et al. 2010; Cox et al. 2015). Photosystem II (PSII), one of the major protein complexes involved in oxygenic photosynthesis, is the catalyst that enables the splitting of two water molecules, whereby four electrons and four protons are removed from water (which are later used for generating chemical energy carriers) and O_2_ is produced as a side-product (Dau and Zaharieva 2009; Vinyard and Brudvig 2017; Junge 2019; Lubitz et al. 2019; Cox et al. 2020; Shevela et al. 2023). PSII has been the subject of many research studies dating back as far as the 1970s, and the main principles of its reaction cycle have been well-established.

**Fig. 1:**
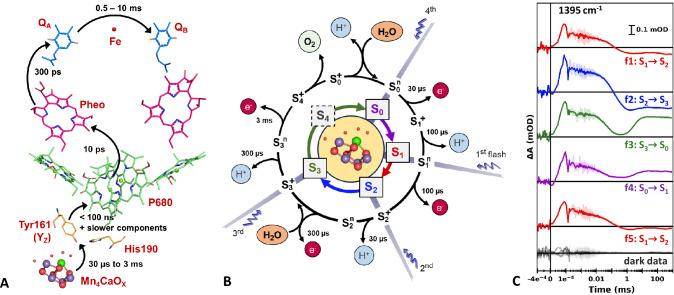
Light-driven reaction cycle of Photosystem II. **A** Co-factors involved in the electron transfer pathway. The absorption of a photon leads to charge separation at the primary donor chlorophylls (P680). The electron moves via a pheophytin (Pheo) to plastoquinone Q_A_ and on to plastoquinone Q_B_. A redox-active tyrosine residue (denoted as TyrZ or Y_Z_) reduces P680^+^ and is in turn reduced by the Mn_4_CaO_x_ cluster, which accumulates oxidative equivalents over the course of four excitation events. **B** Extended S-state cycle scheme, depicting the oxidation state (subscript) and net charge state (superscript) of the Mn_4_CaO_x_ cluster (S_0_ denoting the most reduced state). Starting in the dark-stable S_1_ state, four sequential excitation flashes lead to alternating removal of electrons (e^−^) and protons (H^+^), resulting in the release of molecular oxygen (O_2_) after the third flash. During the S_2_ → S_3_ and S_3_ → S_0_ transitions substrate water molecules are inserted in the first coordination sphere of Mn ions. **C** Exemplary time-resolved IR transients of spinach PSII membrane particles at 1395 cm^−1^ for five sequential excitation flashes. Transients associated mainly with the S_1_ → S_2_ transition is shown in red, S_2_ → S_3_ in blue, S_3_ → S_0_ in green, and S_0_ → S_1_ in purple. The transparent lines show the original data; the darker lines show the same data after applying a smoothing algorithm (sliding average). Four transients, which were acquired in the absence of an excitation flash, are shown in black, illustrating the noise level. The data for t > 0 is shown on a logarithmic scale, while the IR signal over a period of 400 ns before the excitation flash are shown on a linear scale. The time constants indicated in (**A**) and (**B**) are approximate room-temperature values. The scheme in (**A**) and the Mn_4_CaO_x_ cluster in the center of (**B**) were rendered in PyMOL using crystallographic data by Umena et al. (2011) (PDB ID: 3wu2). The hydrocarbon tails of some co-factors in (**A**) have been omitted for simplicity

Following the absorption of a photon, charge separation is initiated at the primary electron donor chlorophylls (P680) involving electron transfer to a neighboring pheophytin molecule (Pheo), thereby generating the charge separated pair P680^⋅+^/Pheo^⋅–^ (for simplicity the symbol indicating the radical character of the groups will be omitted in the following). A series of electron transfer steps (depicted in Fig. 1A) leads to the oxidation of the Mn_4_CaO_x_ cluster, which together with its surrounding amino acid residues and specific protein-internal water molecules comprises the oxygen evolving complex (OEC). Over a series of four absorption events, the OEC accumulates the oxidative power required for the water splitting reaction by going through its so-called S-state cycle (Fig. 1B) (Kok et al. 1970). Beginning in the dark-stable S_1_ state (S_0_ to S_4_, the subscript denoting the number of accumulated oxidizing equivalents), electrons and protons are removed alternatingly, thus keeping the overall charge (and redox potential) of the OEC stable (Dau and Haumann 2007; Klauss et al. 2012a). The final electron removal in the S_3_ → S_4_ transition likely does not result in oxidation of a manganese as in all other transitions, but rather directly in the oxidation of an oxygen atom (Greife et al. 2023), but see also (Shevela et al. 2023).

The events at the OEC are preceded by several important electron transfer steps (Dau and Zaharieva 2009). On the electron acceptor side of PSII, charge separation is further stabilized by transferring the electron from Pheo^–^ to a plastoquinone molecule, Q_A_, within about 300 ps (Nuijs et al. 1986; Eckert et al. 1988). The electron remains at Q_A_ (Q_A_ stays reduced) for 0.5 to 10 ms, before being transferred to a second plastoquinone, Q_B_, which unlike Q_A_ is not tightly bound to the protein. After having taken up two electrons (from two separate primary charge separation events), Q_B_ disassociates from PSII (carrying the electrons to other photosynthetic proteins) and is replaced by an oxidized Q_B_ molecule.

On the electron donor side of PSII, the redox-active tyrosine Y_Z_ has the important task of bridging the gap between P680 and the Mn_4_CaO_x_ cluster. Around 50 years ago, Y_Z_ was still a mystery to be solved. Babcock and Sauer (1975) investigated an EPR signal named *Signal II*_*fast*_, which was only later identified as originating from Y_Z_, as reviewed by Styring et al. (2012). Despite the fact that the redox partner(s) had yet to be identified, it was then already observed that P680^+^ reduction kinetics are multiphasic and S-state dependent (Gläser et al. 1976). Subsequent studies confirmed this, revealing P680^+^ reduction kinetics spanning over four decades from tens of nanoseconds to hundreds of microseconds (Brettel et al. 1984; Schlodder et al. 1985). A slow S-state independent 200 µs phase was attributed to P680^+^/QA^–^ recombination (Schlodder et al. 1985; Christen et al. 1998), resulting in an unsuccessful S-state transition. A fast nanosecond phase of about 20–60 ns was found to be more pronounced in the S_0_ and S_1_ states than in the S_2_ and S_3_ states, and some studies furthermore reported slightly slower time constants for the latter two states (Brettel et al. 1984; Meyer et al. 1989; Haumann et al. 1997; Ahlbrink et al. 1998; Jeans et al. 2002). It was found that this phase showed a relatively low activation energy of about 100 meV (Eckert and Renger 1988) and virtually no sensitivity to H_2_O/D_2_O exchange (Haumann et al. 1997; Ahlbrink et al. 1998; Schilstra et al. 1998). It was proposed (Eckert and Renger 1988) and later discussed in more detail (Christen and Renger 1999; Renger 2004) that this fast nanosecond phase could be linked to the fast movement of a proton between His190 and Y_Z_, but is kinetically limited by an electron transfer (hence the lack of H/D kinetic isotope effect).

A slower nanosecond phase of about 100–800 ns was observed to be clearly more pronounced (Eckert and Renger 1988; Meyer et al. 1989; Lukins et al. 1996; Ahlbrink et al. 1998; Schilstra et al. 1998; Jeans et al. 2002) or even exclusively found (Brettel et al. 1984; Klauss et al. 2012b) in the S_2_ and S_3_ states. H_2_O/D_2_O exchange experiments again showed no kinetic isotope effect (Haumann et al. 1997; Ahlbrink et al. 1998; Schilstra et al. 1998), but the activation energy (∼250 to 300 meV) was found to be higher than for the fast nanosecond phase (Jeans et al. 2002; Kühn et al. 2004; Klauss et al. 2012b). This slow nanosecond phase was interpreted as a local “dielectric” relaxation process (Renger 2004) and later also as nuclear rearrangements resulting in a contraction of about 50 Å^3^ (Klauss et al. 2012b).

Besides the S-state independent 200 μs phase, two further microsecond phases of about 1–8 and 20–40 μs were reported, which were sensitive to H_2_O/D_2_O exchange and mostly exhibited strongest contributions in the S_2_ and S_3_ states (Schlodder et al. 1985; Eckert and Renger 1988; Lukins et al. 1996; Christen et al. 1998, 1999; Schilstra et al. 1998; Christen and Renger 1999). The microsecond kinetics have been interpreted as “large scale proton relaxation” events (Renger 2004). In Mn-depleted PSII samples the nanosecond kinetics are mostly absent and the P680^+^ reduction kinetics are dominated by multiphasic microsecond phases (Haumann et al. 1997; Hays et al. 1999).

To gain insight into their structural environment, P680 and Y_Z_ have also been investigated with FTIR spectroscopy in inactive PSII samples (Zhang et al. 1997; Berthomieu et al. 1998; Noguchi et al. 1998). P680^+^/P680 difference spectra confirmed that the radical cation charge of P680^+^ is mainly localized on one chlorophyll (Okubo et al. 2007; Nagao et al. 2017), which is of significance as the charge localization impacts the redox potential (Takahashi et al. 2008). The P680^+^/P680 difference spectra show a very broad spectral feature ranging from about 1000–6000 cm^−1^ assigned as an intervalence band (Okubo et al. 2007; Noguchi 2010) analogously a similar feature in bacterial reaction centers (Breton et al. 1992) and photosystem I (Breton et al. 1999), this broad band was attributed to an electronic transition originating from the dimeric nature of P680^+^.

More recently FTIR has also been widely applied to intact PSII samples to observe the events of the S-state cycle, as reviewed in (Debus 2015; Noguchi 2015). With the exception of one extremely time-costly step-scan experiment (Greife et al. 2023), these studies are either steady-state measurements or have a time resolution limited to several milliseconds. In an alternative approach, time-resolved IR spectroscopy at single wavenumbers has been employed to observe PSII kinetics at the cost of spectral coverage (Noguchi et al. 2012; Takemoto et al. 2019; Mäusle et al. 2020). The flash-induced transients show distinctly different behavior after each of a sequence of flashes (Fig. 1C), demonstrating that kinetics related to the individual S-state transitions can be readily observed. P680^+^ reduction kinetics have also been previously observed at 4000 cm^−1^ with microsecond time resolution (Sakamoto et al. 2017). By acquiring transients at individual wavenumbers over a large spectral region, we recently showed for PSI that we can obtain a time-resolved spectral data set in a relatively short measurement time with good a signal to noise ratio (SNR) (Mäusle et al. 2023).

We here report the first IR measurements of P680^+^ reduction kinetics with sub-microsecond time resolution in intact oxygen evolving PSII membrane particles from spinach. By measuring the time-resolved IR difference signal at wavenumbers associated only with the broad electronic band (> 1760 cm^−1^), we can observe the events at P680 without contributions of other groups. Double-difference spectra on the early-microsecond time scale of a time-resolved spectral data set furthermore allow us to obtain approximate P680 Yz^ox^/ P680^+^ Yz spectra in the 1310–1760 cm^−1^ region.

## Materials and methods

### Sample preparation

PSII membrane particles were prepared from spinach leaves as described previously (Schiller and Dau 2000), based on a protocol developed by Berthold, Babcock, and Yocum (Berthold et al. 1981). The oxygen evolution activity (> 1100 μmol O_2_ per mg chlorophyll and hour) was measured with a Clarke-type electrode at 28 °C using 10 μg of Chl in 1 M betaine, 25 mM MES, 15 mM NaCl, 5 mM CaCl_2_ (pH 6.2) buffer, 1 mM K_3_[Fe(CN)_6_] and 0.25 mM DCBQ (2,6 dichloro-1,4-benzoquinone), of which the latter two ingredients serve as artificial electron acceptors. The sample was stored in 1 M betaine, 25 mM MES, 15 mM NaCl, 5 mM M_g_Cl_2_, 5 mM CaCl_2_ (pH 6.2) at – 80 °C. In preparation for an IR measurement (corresponding to 3 mg Chl) the sample was thawed on ice for an hour before resuspension in the measurement buffer (identical to the storage buffer) and centrifuging for 12 min at 50.000×*g*. The supernatant was discarded and the same resuspension-centrifugation washing step repeated one more time. An artificial electron accepter PpBQ (phenyl-*p*-benzoquinone, 3 μl of 700 mM in dimethyl sulfoxide) was mixed with the resulting pellet. 10–12 mg of the pellet was squeezed between two CaF_2_ plates with a 15 μm PTFE spacer; silicon grease was used on the edges of the plates for sealing. Per measurement day, 4–5 pairs of CaF_2_ plates were mounted onto the automatic sample-exchange stage. All the above-mentioned steps were done under dim green light, otherwise the sample was kept in the dark.

### IR measurements

The IR transients were measured using a quantum cascade laser (QCL) based time-resolved single-frequency (TRSFIR) setup described previously (Mäusle et al. 2020) which has since been modified as described elsewhere (Mäusle et al. 2023). The QCL (MIRcat-QT-Z-2300, Daylight Solutions, USA) is tuneable in the range between 1310 to 1890 cm^−1^. A 5 ns frequency-doubled 532 nm Nd-YAG laser (Minilite II, Continuum, USA) was used for excitation and the signal from two 10 MHz pre-amplified mercury cadmium telluride (MCT) detectors (Vigo Systems, Poland) was recorded using 16-bit A/D converter (Spectrum, Germany) at a sampling rate of 65 MS/s. The sample compartment was cooled to 10 °C and flushed with dry air.

To synchronize the PSII membrane particles to the same S-state (S_1_) before beginning the measurements, two saturating pre-flashes were applied to all sample spots, followed by an hour of dark-adaptation. For each of ∼200 individual sample spots, the absorbance of the dark-adapted BBY sample was measured for a few seconds before a series of 10 saturating flashes at 1 Hz were applied. Following each 10-flash burst, the x–y-movable sample holder moved to a new dark-adapted sample spot. All transients are affected by a heat artefact, which was accounted for by acquiring the 10th flash IR transient with a threefold higher excitation energy and subsequently calculating and subtracting the heat signal as described previously (Mäusle et al. 2020), similar to an approach reported by Sakamoto et al. (2017). Time-resolved spectral information was obtained by performing the measurement sequentially at individual wavenumbers along the QCL range, in steps of 2 cm^−1^. The final transients for individual wavenumbers were obtained by averaging of at least 20 transients. Originally 290 wavenumbers were measured between 1310 and 1890 cm^−1^. Only 276 wavenumbers were used in the final spectra presented in this work, where the rest were omitted due to poor data quality. As the SNR of the individual transients was overall limited due to a low number of averages per transient, applying a sliding average smoothing algorithm (with a window size of three data points) along the wavenumber axis was necessary to obtain reasonably smooth spectra; this resulted in an effective spectral resolution of about 6–8 cm^−1^.

Transients were fit in the range from 0 to 100 μs to a model defined by a sum of four exponentials parametrized as follows:

1 $$y\left(t\right)={y}_{o}+{a}_{1}{e}^{-t/{\tau }_{1}}+{a}_{2}{e}^{-t/{\tau }_{2}}+{a}_{3}{e}^{-t/{\tau }_{3}}+{a}_{4}{e}^{-t/{\tau }_{4}}.$$

Additionally, an instrument response function (IRF) was taken into consideration which was iteratively convolved with the multi-exponential model during least-squares optimization (Python 3.9.2 and lmfit 1.2.2), as detailed in Sect. 1 of the SI. The IRF was approximated as

2 $${\text{IRF}}\left(\tau \right)={e}^{\frac{{-\left(\tau -{\tau }_{o}\right)}^{2}}{{2\sigma }^{2}}},$$

with σ = 17 ns (Mäusle et al. 2023) and *t*_0_ = 44 ns. All data pre-processing was performed in Python 3.7.

## Results

### IR difference spectra related to P680^+^/P680, Y_Z_^ox^/YZ and QA^−^/QA

To investigate the kinetics preceding the events of the S-state cycle, we applied sequences of 10 saturating laser flashes to dark-adapted PSII membrane particles at ∼276 individual wavenumbers ranging from 1310 to 1890 cm^−1^ (with a spacing of mostly 2 cm^−1^). The flash-induced absorption changes were recorded in the time range from nanoseconds to 800 ms; see Fig. 1C for exemplary high signal-to-noise data. From this data set, difference spectra can be constructed that correspond to distinct times after the respective laser flashes. In the present study, we focus on the spectra and reaction kinetics associated with fast P680^+^ formation and its reduction by the redox-active tyrosine, Y_Z_. In PSII with an intact oxygen-evolving complex, the latter is largely completed within 10 µs after the laser flash. Therefore, we focus on the events proceeding within 10 µs after the laser flash.

**Fig. 2:**
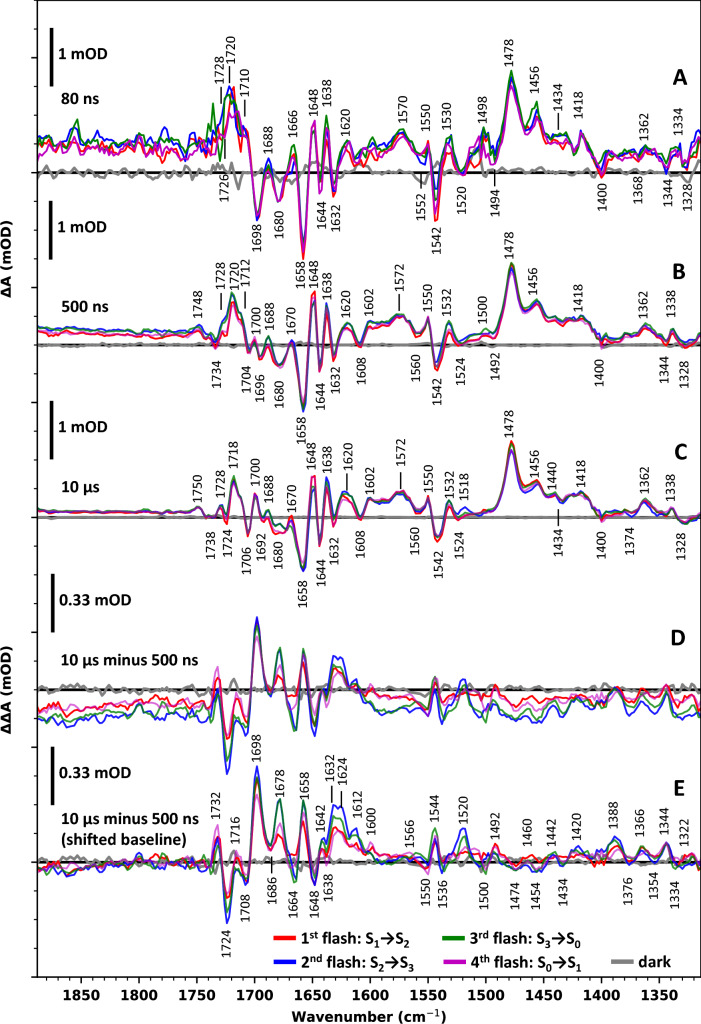
Flash-induced time resolved IR difference spectra of spinach PSII membrane particles at 10 °C. The difference absorbance (ΔA) was obtained at every ∼ 2 cm^−1^ between ∼1310 and 1890 cm^−1^ by acquiring flash-induced transients; a sliding average over 3 neighboring wavenumber was applied, resulting in an effective spectral resolution of 6–8 cm^−1^. Data associated mostly with the S_1_ → S_2_ transition (first flash) is shown in red, the S_2_ → S_3_ transition (second flash) in blue, the S_3_ → S_0_ transition (third flash) in green and the S_0_ → S_1_ (fourth flash) in magenta; the noise level is illustrated by 'dark spectra' (grey), obtained in the same way as the flash-induced data, but without applying an excitation flash. **A** IR difference spectra of around 80 ns after the first four excitation flashes. The spectra were obtained by averaging the data acquired between 50 and 110 ns. **B** IR difference spectra of around 500 ns (250–750 ns) after excitation. **C** IR difference spectra of around 10 μs (8–12 μs) after excitation. **D** Double difference spectra of the 10 μs spectra minus the 500 ns spectra (spectra C–spectra B). The resulting double difference absorption (ΔΔA) was small compared to the data shown in **A**, **B**, and **C** and was thus multiplied by a factor of 3 for better visualization (as indicated by the scale bar). **E** A baseline-corrected version of the double difference spectra shown in **D**, i.e. the spectra of 10 μs minus 500 ns. The non-smoothed spectral data (Fig. S3) as well as details of the two-step baseline correction can be found in Supplementary Information

Difference spectra of around 80 ns (50–110 ns average) after the laser flash are shown in Fig. 2A, around 500 ns (250–750 ns) in Fig. 2B, and around 10 μs (8–12 μs) in Fig. 2C. All spectra are shown for the first four flashes applied to dark-adapted PSII, each of the four flashes initiating predominately one specific S-state transition, starting with S_1_ → S_2_ (see also Fig. 1B). The spectra shown in Fig. 2 relate to the events following charge separation in PSII as follows:

(A) At 80 ns after the laser flash, the difference spectra reflect predominantly the formation of P680^+^ and Q_A_^−^ (Fig. 2A).
(B) In Fig. 2B, the difference spectra at about 500 ns are shown. At this time, the fast nanosecond phase of P680^+^ reduction and Y_Z_ oxidation has resulted in strongly diminished P680^+^/P680 contribution, whereas the amplitude of the Q_A_^−^/Q_A_ difference can be expected to be undiminished when compared to the 80 ns spectra of Fig. 1A.
(C) At 10 µs, the P680^+^ reduction is mostly completed so that the corresponding difference spectra in Fig. 2C are composed of the Y_Z_^ox^/Y_Z_ and Q_A_^−^/Q_A_ difference spectra. The Q_A_^−^/Q_A_ contribution is expected to be undiminished when compared to the 80 ns spectrum.
(D) Figure 2D shows the double-difference absorbance (ΔΔA) of the 10 μs spectrum minus the 500 ns spectrum (C minus B). The ΔΔA spectrum corresponds exclusively to P680^+^ reduction and Y_Z_ oxidation and thus represents the sum of Y_Z_^ox^/Y_Z_ and P680/P680^+^ difference spectra, without contributions from the Q_A_^−^/Q_A_ difference spectrum. (Note that P680^+^/P680 peaks visible in A and B are inverted in the double difference spectra of D and E).
(E) Figure 2E shows the same spectrum as shown in D but corrected for the time-dependent background shift, as detailed in Supplementary Information. For baseline correction, one constant was determined for each of the four spectra and then subtracted from all ΔΔA values of the respective spectrum. This procedure corresponds to subtraction of a baseline that is parallel to the x-axis. It represents an approximative approach here used to improve visibility of positive/negative peaks in the spectra and S-state dependent differences between spectra.

When comparing the S-state dependence (flash-number dependence) of the spectra in Fig. 2, we note minor difference in shape only but detect significant differences in the amplitude at early times. The amplitudes of positive and negative peaks are often larger in the second and third flash spectra than in the first and fourth flash spectra. A broad positive feature visible above 1750 cm^−1^ effectively results in a background shift of the spectra. This broad feature is most prominent in the early spectrum (Fig. 2A) and subsequently decays to smaller contributions, as can be easily observed by visually comparing Fig. 2A–C.

Comparing Fig. 2A–C, the positive peaks at 1362, 1418, around 1440, 1456, 1478 cm^−1^ and the negative peak at 1644 cm^−1^ show no clear change in their intensities over time, which is the expected behavior of quinones (see above). In addition, they show merely a weak or no presence in the double difference spectra (Fig. 2D and E). Consequently, we assign these peaks to the oxidized (Q_A_, negative peak) or the singly reduced quinone (Q_A_^−^, positive peaks), in agreement with previous assignments (Hienerwadel et al. 1996; Zhang et al. 1997; Berthomieu et al. 1998; Suzuki et al. 2005). We detect further peaks likely related to the Q_A_^−^ formation at 1328 (−), around 1572 (+), 1658 (−), and 1718 (+) cm^−1^.

A negative peak at 1698 cm^−1^ (Fig. 2A) possibly downshifted to 1696 and 1692 cm^−1^ in Fig. 2B and C, respectively, displays a strong decrease in intensity over time. Although visually harder to detect because of the upshifted baseline, the negative peaks at 1552 and 1344 cm^−1^ exhibit a similar decaying behavior. All three features, however, show a clear positive band in the baseline-corrected double difference spectrum (Fig. 2E) and resemble peaks previously assigned to the 13^1^-Keto C=O and chlorin ring vibrations of neutral P680 (Okubo et al. 2007). The positive signal around 1724 cm^−1^ (Fig. 2A) decays to around zero (Fig. 2C), resulting in a distinctly negative peak in Fig. 2E; a similar behavior is observed for the signal around 1708 cm^−1^. These two bands resemble a doublet feature assigned to the P680^+^ cation, arising from the uneven charge distribution across the two primary donor chlorophylls (Okubo et al. 2007; Nagao et al. 2017).

Three positive peaks at 1638, 1648, 1688 cm^−1^ in Fig. 2A also exhibit a decrease in intensity in Fig. 2B and C, resulting in prominent negative peaks in the double-difference spectra (Fig. 2E). The decaying negative peaks at 1680 and 1658 cm^−1^ result in positive peaks in Fig. 2E. A negative band at 1734 cm^−1^ in Fig. 2B that upshifts to 1738 cm^−1^ in Fig. 2C displays a less clear behavior, but nevertheless results in a prominent positive peak in Fig. 2E. All the aforementioned peaks have counterparts in previously reported P680^+^/P680 spectra (Nagao et al. 2017). In fact, the entire region between 1732 and 1520 cm^−1^ in Fig. 2E strongly resembles the shape of P680/P680^+^ spectra (with some differences in the relative intensities of the peaks).

As outlined above, Y_Z_^ox^/Y_Z_ features are expected to be mostly absent in the 80 ns spectrum and to become more prominent at later time points. Y_Z_^ox^ features should thus appear as positive contributions (positive peaks) in Fig. 2E, while Yz features should be negative. Comparing the signal amplitudes reported for P680^+^/P680 and Y_Z_^ox^/Y_Z_, it is evident that Y_Z_^ox^/Y_Z_ signals are roughly ten times less pronounced (Berthomieu et al. 1998; Nagao et al. 2017). However, the previously small amplitudes in the Y_Z_^ox^/Y_Z_ difference spectrum also could relate to formation of Y_Z_^ox^ in a comparably small fraction of PSII only. Previously, prominent positive peaks at about 1512 and 1550 cm^−1^ alongside a negative peak at 1543 cm^−1^ have been reported (Nakamura et al. 2014; Nagao et al. 2017), which does not agree well with features in Fig. 2E. For the second and third flash difference spectra, we detect a positive peak at 1520 cm^−1^, whereas for the first and fourth flash spectra a broad (but weak) peak around 1512 cm^−1^ may be present, possibly suggesting that the presence of an intact OEC modifies the spectral features previously reported for the Y_Z_^ox^/Y_Z_ difference spectrum in Mn-depleted PSII.

The features in the spectral region of Fig. 2E overall show a better agreement with features related to the P680^+^/P680 difference spectrum. The triplet-shaped peak typically observed in Y_Z_ spectra around 1677–1700 cm^−1^ is also not visible in Fig. 2E. It is, however, faintly visible in Fig. 2B: when comparing Fig. 2B and C (while keeping in mind the baseline shift), the positive peaks at 1700 and 1688 cm^−1^ are clearly increasing, while a new peak at 1680 cm^−1^ forms—but the latter only in the third flash spectrum. The derivative-like group of peaks at 1550, 1542 and 1532 cm^−1^ in Fig. 2B and C also resemble previous findings in Y_Z_^ox^/Y_Z_ spectra; but these peaks are absent or inverted in Fig. 2D and E.

In experiments on Mn-depleted PSII particles, a differential signal of the Y_Z_^ox^/Y_Z_ difference spectrum has been detected at ca. 1700/1706 cm^−1^ (positive/negative peak) and assigned to perturbation of the keto C=O vibration of the P680 chlorophyll denoted as PD1 (Berthomieu et al. 1998; Nakamura et al. 2014; Nagao et al. 2017). This differential 1706/1700 cm^−1^ feature is well resolved also in the spectra of Fig. 2C (collected at 10 µs; negligible P680^+^ population), in line with assignment to Y_Z_^ox^/Y_Z_ and confirming the results previously reported for Mn-depleted PSII. The double-difference spectra of Fig. 2E, however, reflect a more complex situation. In Fig. 2E, not only the Y_Z_^ox^/Y_Z_ features at 1700 and 1706 cm^−1^ of Fig. 2C contribute to the peaks detected at 1708 and 1698 cm^−1^, but also prominent features of the P680^+^/P680 difference spectrum. Comparison of the time evolution of the 1708 cm^−1^ and 1698 cm^−1^ IR signals with the P680^+^ population (which is analyzed further below) suggests that the P680^+^/P680 feature provides the more major contribution to the 1708/1698 cm^−1^ peaks in Fig. 2E. Also the presence of a strong negative peak at 1698 cm^−1^ in the 80 ns spectrum and the relative intensities of the 1708/1698 cm^−1^ peaks support a dominating contribution of the P680/P680^+^ difference spectrum. We conclude that the double-difference spectrum in Fig. 2E is largely dominated by P680/P680^+^ contributions, although Y_Z_^ox^/Y_Z_ features also contribute.

In light of the above conclusion, one may take a closer look at differences between Fig. 2E and the P680^+^/P680 difference spectra in previous FTIR studies on Mn-depleted and thus O_2_-inactive PSII sample. While here we see a prominent peak at 1544 cm^−1^, this peak only appears as a small side-peak in previous reports for P680^+^/P680. A previously reported peak at 1557 cm^−1^_,_ which should appear as positive in Fig. 2E, is on the other hand missing. Furthermore, the peak around 1633 cm^−1^ is much broader in our data and exhibits a clear S-state dependence, clearly indicating an influence of the OEC (and/or its environment) on these fast processes.

### P680^+^ reduction kinetics observed around 1800 cm^−1^

**Fig. 3:**
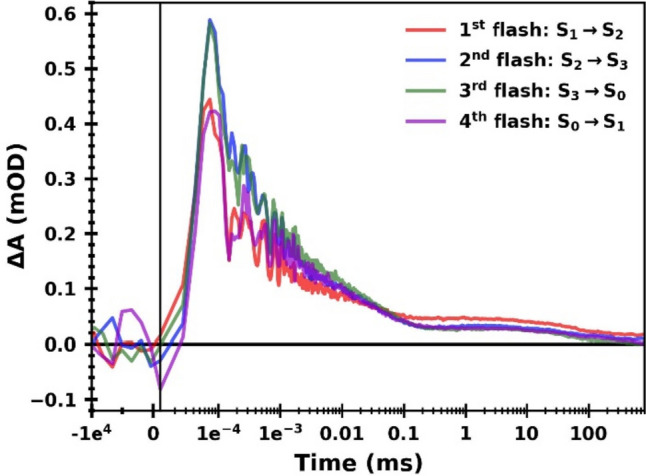
Flash-induced IR difference absorption at wavenumbers greater than 1760 cm^−1^, corresponding to the broad time-dependent background visible in the spectra in Fig. 2. All transients acquired between 1760 and 1884 cm^−1^ were averaged to improve the signal-to-noise ratio. Data associated mostly with the S_1_ → S_2_ transition (first flash) is shown in red, the S_2_ → S_3_ transition (second flash) in blue, the S_3_ → S_0_ transition (third flash) in green and the S_0_ → S_1_ (fourth flash) in magenta. The signal from 100 ns before the excitation flash up to 11.5 ns after the flash is shown on a linear scale, while the data between 11.5 ns and 800 ms is shown on a logarithmic x-axis; the two different axes are separated by a vertical line

Figure 3 shows the time-resolved IR difference signal following the first four excitation flashes, obtained by averaging the signal at 51 individual wavenumbers in the range of 1760–1884 cm^−1^. As seen in Fig. 2, the region above 1760 cm^−1^ exhibits a background (baseline) shift but no prominent features; the transients of the individual wavenumbers all display similar kinetics (data not shown) and were thus averaged to reduce the noise level. The four flash-induced transients in Fig. 3 show overall very similar kinetics. A rise to the maximum signal, which is higher after the second and third flash than after the first and fourth flash, occurs within about 80 ns (the delay to reaching the maximum is merely due to slow instrument response). All four transients show a multiphasic decay back to zero, whereby the first and fourth as well as the second and third flash transients show near identical behavior to each other. At around 100 µs, any S-state dependence disappears; merely the first flash transient shows a slightly up-shifted amplitude in the range from ∼ 100 µs to 800 ms. The fifth flash, as well as all following flash-induced transients, do not show this upshifted signal (Fig. S4), which is thus attributed to a signal contribution of presently unknown origin exclusive associated with the first flash applied to dark-adapted PSII.

**Fig. 4:**
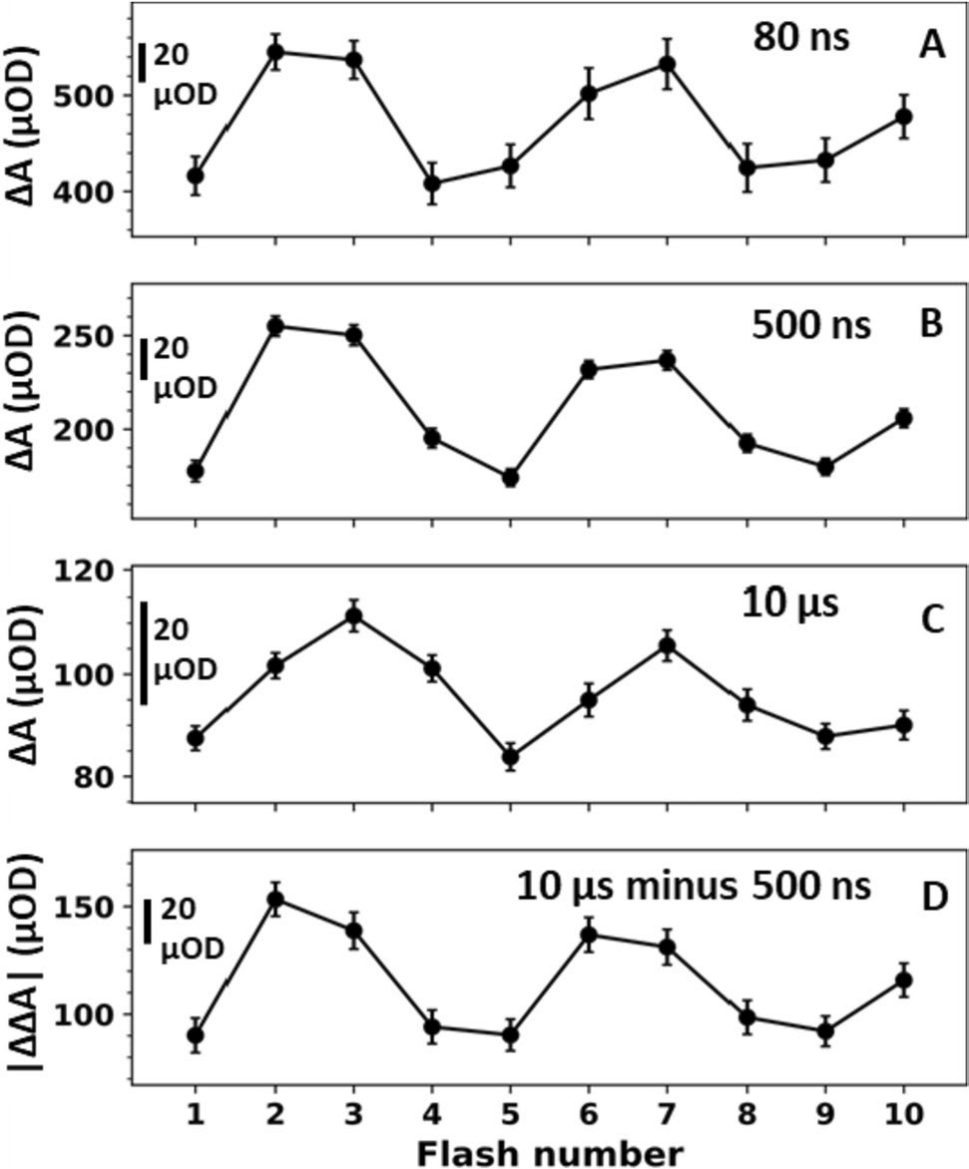
Averaged IR difference absorption (ΔA) at wavenumbers greater than 1760 cm^−1^ as a function of flash number at different times after the respective excitation flash. The data shown here corresponds to the data set shown in Figs. 3 and S4. **A** IR difference absorption averaged around 80 ns (50–110 ns). **B** IR difference absorption averaged around 500 ns (250–750 ns). **C** IR difference absorption averaged around 10 µs (8–12 µs). **D** Absolute IR double difference absorption obtained by subtracting the signal around 500 ns from the signal around 10 µs (**C** minus **B**, multiplied by − 1). All averaged IR absorption values (**A**–**D**) exhibit a clear period-of-four dependency on the flash number. The error bars correspond to the standard error obtained from averaging the signal at all wavenumbers between 1760 and 1884 cm^−1^

Figure 4A–C show the averaged difference absorption as a function of flash number for the data shown in Fig. 3 (and its subsequent six flashes, shown in Fig. S4) at around 80 ns, 500 ns and 10 µs, respectively. Figure 4D additionally shows the absolute values at 10 µs minus 500 ns. All four flash-number dependent IR signals show a clear quaternary oscillatory pattern with a maximum either at the second flash (Fig. 4A, B, D) or at the third flash (Fig. 4C).

**Fig. 5:**
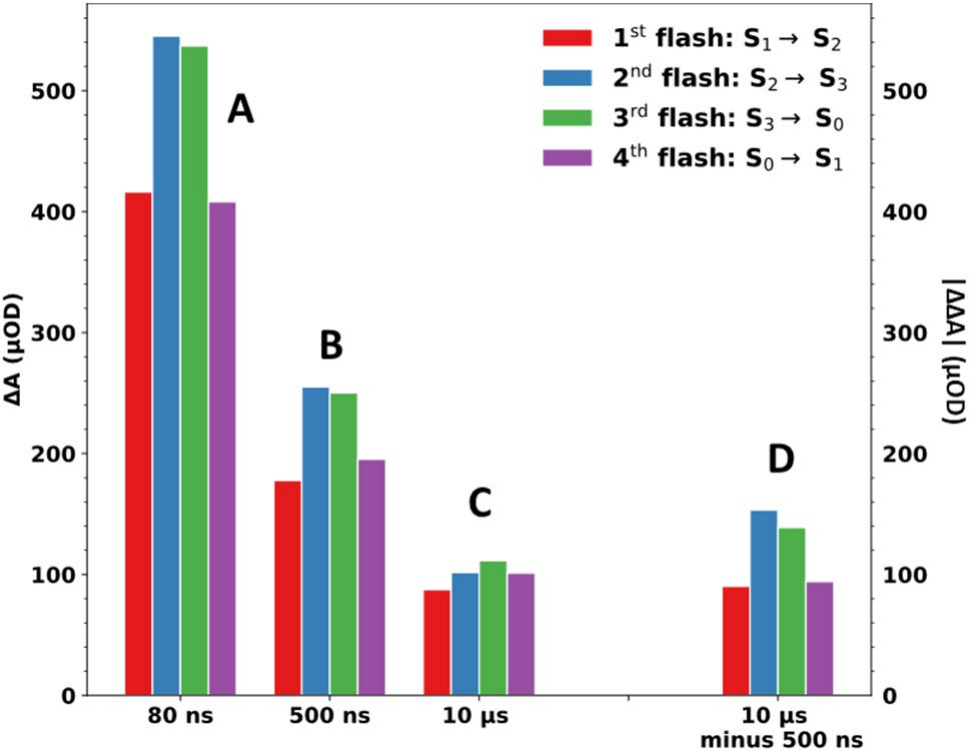
Bar plot of the averaged IR difference absorption (ΔA) at > 1760 cm^−1^ at different time points following the first four excitation flashes, indicating the time- and S-state-dependent background signal of the spectral data in Fig. 2. The data shown here corresponds to the data set shown in Fig. 3 (average of all transients between 1760 and 1884 cm^−1^). Data associated mostly with the S_1_ → S_2_ transition (first flash) is shown in red, the S_2_ → S_3_ transition (second flash) in blue, the S_3_ → S_0_ transition (third flash) in green and the S_0_ → S_1_ (fourth flash) in magenta. The IR difference absorption was averaged around **A** 80 ns (50–110 ns), **B** 500 ns (250–750 ns) and **C** 10 µs (8–12 µs). The absolute IR double difference absorption obtained by subtracting the signal around 500 ns from the signal around 10 µs (**C** minus **B**, multiplied by − 1) is shown in (**D**)

**Fig. 6:**
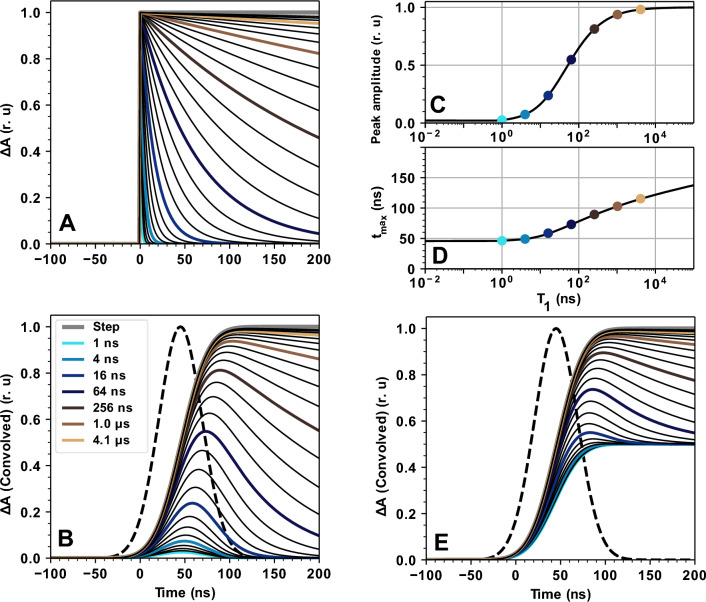
Influence of a limited temporal resolution of the experimental set up, represented by a Gaussian instrument response function, on the maximal (peak) intensity and peaking time of a hypothetical P680^+^ absorption transient. **A** Ideal absorption transients immediate P680^+^ formation and complete P680^+^ re-reduction by a single-exponential decay, with a hypothetical decay time varied between 1 ns and 4.1 µs (colored lines). Additionally, a hypothetical stable P680^+^ formation (step function) is shown as a grey line. Thinner black lines indicate transients with components that lie in between the rates of neighboring color-coded transients (the decay time of neighboring curves differ by a factor of $\sqrt{2}$). **B** The transients shown in **A** convolved with a Gaussian instrument response function (IRF) with a standard width of 17 ns (dashed line) which was determined previously (Mäusle et al. 2023). **C** Influence of the P680^+^ decay time constant on the maximal amplitude (peak amplitude) of the convolved transients of **B**. **D** Influence of the P680^+^ decay time constant on the time of the maximal signal amplitude (t_max_). **E** Absorption transients simulated by two exponential components convolved with the Gaussian IRF. One exponential is set to a very slow value, mimicking a step-response, while the faster exponential is varied in the same way as in **A** and **B** (1:1 amplitude ratio of fast and slow component). In **C** and **D** the colored dots represent the respective transients of **B**. In all panels the colors indicate the (fast) decay constants given in the legend shown in **B**

This period-of-four behavior of the 1884–1760 cm^−1^ data, together with the well-established notion that a broad electronic band associated with P680^+^ extends across the entire range from 1000 to 6000 cm^−1^ spectral region (Okubo et al. 2007), provides a strong indication that the transients in Fig. 3 are directly associated with P680^+^ reduction kinetics of intact, oxygen-evolving PSII. The flash-number dependence (and thus S-state dependence) of the P680^+^ background signal at 80 ns, 500 ns and 10 µs (as well as 10 µs minus 500 ns) is shown as a bar diagram in Fig. 5. All trends visible in Fig. 5 reflect a real S-state dependence because they are in line with the respective oscillatory pattern of Fig. 4. On a first glance, the S-state dependence of the maximal P680^+^ level detected at 80 ns is surprising because each of the saturating laser flashes is expected to induce P680^+^ formation in essentially all PSII centers, suggesting the same initial P680^+^ level. This behavior of the here detected P680^+^ signal is traced back to the limited time resolution of our experiment. It is explainable by a faster P680^+^ decay time on the first and fourth flash than on the second and third flash, as illustrated by simulations of hypothetical P680^+^ transients shown in Fig. 6.

**Fig. 7:**
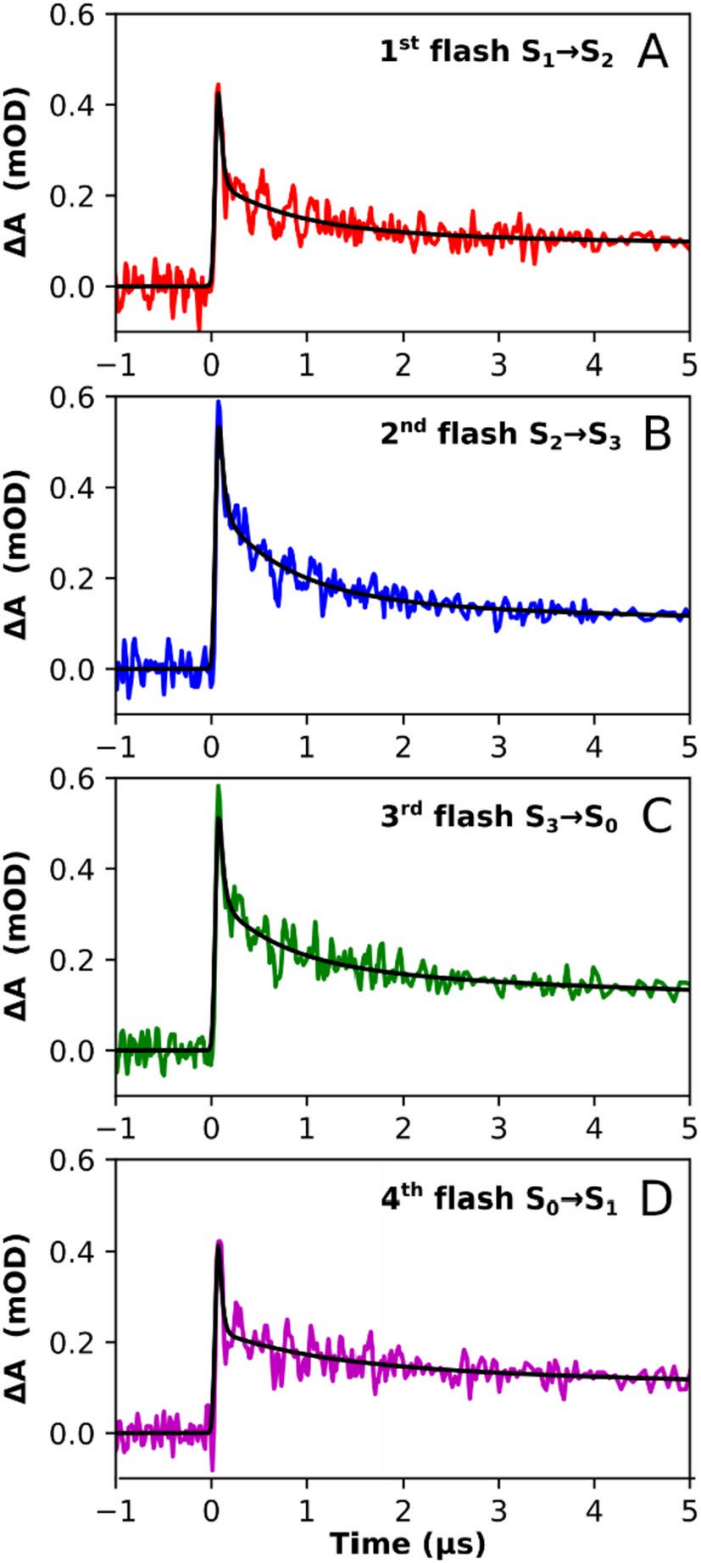
Multi-exponential fits of the IR transients of wavenumbers greater than 1760 cm^−1^, following the first four excitation flashes. The data (colored lines) corresponds to that of Fig. 3, but here shown with a linear time axis for the times ranging from 1 µs before and 5 µs after the respective laser flash. The colored lines indicate the recorded data while the black lines indicate the simulations obtained by a least-squares fit of the multi-exponential P680^+^ decay that involves convolution with the instrument response function, to account for the limited temporal resolution of the experiment. For each of four transients shown in panel **A**–**D**, the P680^+^ decay was simulated by a sum of four exponentials plus an offset term (y_0_), taking into account the instrument response function (see “Materials and methods” section for details of the fit approach). The fit results, i.e., four amplitudes (a_1_ to a_4_) and four time constants (τ_1_ to τ_4_) for each of the transients shown in **A**–**D**, are presented in Fig. 8

**Fig. 8:**
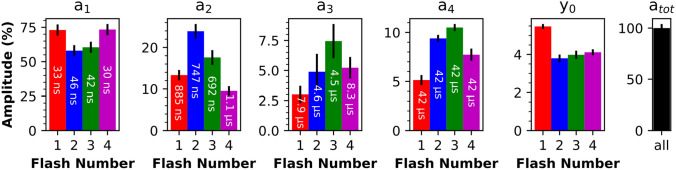
Results of the multi-exponential fit of the first four flash-induced transients at greater than 1760 cm^−1^ (shown in Fig. 7). Each transient was fitted to a sum of four exponentials and an offset (y_0_); the amplitudes are normalized to the sum of all amplitudes plus offset and therefore represent the relative contribution of the four components to the fitted transients. The amplitudes and time constants were free to vary, solely τ_4_ was constrained to be identical in all four transients (fit range from 0 to 100 µs). The amplitudes and y_0_ are shown as bar plots and the respective time constants are indicated. The 1σ uncertainty ranges of the amplitudes, obtained from the covariance matrix provided by the least squares fitting procedure, are indicated by vertical black lines. The absolute values of the amplitudes, as well as the offset, are shown in Fig. S1 and Table S2 of Supplementary Information

To further characterize this P680^+^ background signal, the transients in Fig. 3 were simulated with a sum of four exponentials, taking into account the limited temporal resolution of the experiment (by convolution with the IRF as detailed in the SI). Simulated transients as obtained by least-squares fitting with calculation of the error sum for times ranging from 0 to 100 µs and the corresponding simulation parameters are shown in Figs. 7 and 8, respectively. The slowest time constant was constrained to be the same in all four transients; the other three time constants could vary freely. All amplitudes could vary freely, but the sum of all amplitudes (plus offset) was constrained to be the same in all transients, justified by the assumption that the magnitude of the initial P680^+^ signal is the same in all S-state transitions. In line with the illustrative simulations of Fig. 6, the fit parameters of Fig. 8 show that the S-state dependence of the initial peak heights of the transients in Fig. 3 results from the fastest time constant of the second and third flash transients being slower than the fastest time constants of the first and fourth flash transients.

For all four transients, the values of the fast nanosecond time constant (τ_1_, 30–50 ns)) as well as the corresponding amplitudes (highest in S_1_ and S_0_, lowest in S_2_ and S_3_) are in good agreement with previous results (Karge et al. 1996; Schilstra et al. 1998), inter alia verifying the used approach to consider the time resolution of the experiment in the least-squares curve-fit of the transients. The S-state dependence of the amplitudes of the slower nanosecond component (τ_2_) is also in good agreement with previous findings on P680^+^ kinetics (highest value after the second flash); the value for τ_2_ found for the fourth flash transient (1.1 µs), however, is somewhat slower than values reported previously (Eckert and Renger 1988; Meyer et al. 1989; Lukins et al. 1996; Ahlbrink et al. 1998; Schilstra et al. 1998; Jeans et al. 2002). Both microsecond components, τ_3_ (4–8 µs) and τ_4_ (42 µs), are in line with previous results on P680^+^ reduction kinetics regarding both time constant values and S-state dependence of amplitudes (Christen et al. 1998; Schilstra et al. 1998). In summary, the P680^+^ reduction kinetics determined by analysis of absorption changes in the IR range agree very well with experimental results previously obtained by optical spectroscopy in the visible or near-infrared regime (the latter around 820 nm). Aside from non-identical temperatures during data collections, the differences in simulation parameters likely reflect variations in the used PSII preparations (in particular varying fractions of O_2_-inactive PSII) or relate to details of the multi-exponential fits in the presence of significant noise contributions.

## Discussion

### P680^+^/P680 and Y_Z_^+^/Y_Z_ difference spectra of intact PSII particles

Here we present the first time-resolved investigation on PSII in the mid-infrared regime that addresses the laser-flash induced [P680^+^, Q_A_^−^] radical-pair state and its decay by reduction of P680^+^ by the redox-active tyrosine (Y_Z_) with multi-phasic decay kinetics. Difference spectra for the Q_A_^−^/Q_A_, P680^+^/P680, Y_Z_^+^/Y_Z_ transitions have been reported before based on conventional (not time-resolved) FTIR spectroscopy and sophisticated experimental protocols to ‘isolate’ the difference spectra assignable to reduction (Q_A_^−^/Q_A_) or oxidation (P680^+^/P680, Y_Z_^+^/Y_Z_) of a specific redox-factor (Zhang et al. 1997; Berthomieu et al. 1998; Noguchi et al. 1998; Berthomieu and Hienerwadel 2005; Okubo et al. 2007; Nakamura et al. 2014; Kato et al. 2016; Nagao et al. 2017). For detecting the P680^+^/P680 and Y_Z_^+^/Y_Z_ difference spectra, typically Mn-depleted PSII particles were employed, where the donor side of PSII had been seriously modified by removal of the Mn_4_CaO_x_ cluster, typically in conjunction with removal of some membrane-extrinsic PSII polypeptides. The here reported time-resolved experiments now facilitate the comparison of the P680^+^/P680 and Y_Z_^+^/Y_Z_ difference spectra between Mn-depleted and intact O_2_-evolving PSII.

Overall, the double difference spectra of intact, O_2_-evolving PSII (Fig. 5E) exhibit excellent agreement with the previously reported P680^+^/P680 spectra of Mn-depleted PSII (Okubo et al. 2007) suggesting that the spectral characteristics of both P680 and P680^+^ are not strongly affected by the presence of the Mn_4_CaO_x_ cluster. There are clear similarities with previously reported Y_Z_^+^/Y_Z_ difference spectra of Mn-depleted PSII (Nagao et al. 2017), but also clear differences.

When compared to spectra of Mn-depleted PSII, the clear S-state dependence of many peaks provides new information. For features exclusively related to P680/P680^+^ one might expect them to reflect the S-state dependence of the broad feature P680^+^ detected above 1760 cm^−1^ (Fig. 5D), that is, the peak amplitudes of the second and third flash spectra are expected to be moderately enlarged compared to that of the first and fourth flash. However, some peaks (1678(+), 1664(−), 1630(+) and 1520(+) cm^−1^) show a more pronounced S-state dependence of amplitudes than expected, while others (1708(−), 1544(+), 1366(+), 1344(+) cm^−1^) show, within the noise-level, none. We hypothesize that the latter features could be indirectly related only to P680^+^ and Y_Z_^ox^ formation, e.g. by long-range electric field effects.

Three of the features with strongly S-state dependent amplitudes (1664(−), 1630(+) and 1520(+)) are likely related to Y_Z_^ox^/Y_Z_ (the following comparisons relates to the spectra reported by Nagao et al. (2017)):

(i) The negative feature at 1664 cm^−1^ in the second and third flash spectra is broader and slightly upshifted in the first and fourth flash. Y_Z_^ox^/Y_Z_ spectra of inactive PSII show a strong feature at 1665(−) and 1677(+), which might be the cause of this additional modulation.
(ii) The feature around 1630 cm^−1^ exhibits a small, but clear, double peak in the second and third flash spectra with an additional side peak at 1612 cm^−1^. In contrast, it appears as one broad feature in the first and fourth flash. Y_Z_^ox^/Y_Z_ spectra of inactive PSII show a negative peak at 1627 cm^−1^, which could be the cause of the small dip in our data (giving the feature the appearance of a double peak). The side peak at 1612 cm^−1^ could similarly be caused by the dip around 1620 cm^−1^, which is also present as a negative peak in Y_Z_^ox^/ Y_Z_ spectra of inactive PSII.
(iii) The prominent peak in the second and third flash spectra at 1520 cm^−1^ is replaced by a smaller broad feature in the first and fourth flash. From spectra of inactive PSII, we would expect a 1520(+) peak for P680/P680^+^ and a 1512(+) peak for Y_Z_^ox^/ Y_Z_. The broad feature in the first and fourth flash spectra could be a merged peak of both. Possibly the 1512 cm^−1^ Y_Z_^ox^ peaks upshifts in the second and third flash, resulting in a very pronounced peak at 1520 cm^−1^.

The relations to Y_Z_^ox^/Y_Z_ spectra of Mn-depleted PSII discussed above are still hypothetical, inter alia due to the possible influence of noise contributions, and require further investigation. However, irrespective of the spectral details, the pronounced S-state dependency of several spectra features implies that in future investigation the S-state specific events associated with Y_Z_ oxidation, which could be rearrangement of the protein-water network in the vicinity of Y_Z_ and the OEC as well as conformational changes of the tyrosine residue itself, may be traced by infrared spectroscopy.

### Fast acceptor side processes?

The interpretation of the spectra so far was done under the assumption that acceptor-side kinetics do not contribute significantly to the IR signals in the early time domain. Indeed, the absorption at 1478 cm^−1^, which has been assigned to a semiquinone mode (Berthomieu et al. 1990), is mostly constant between 500 ns and 10 µs after correcting for its P680^+^ background (Fig. S8A). This implies that the quinone oxidation state does not change in this time range. Yet a closer look at other wavenumbers previously assigned to the acceptor-side (1658, 1638, 1552 and 1532 cm^−1^; (Berthomieu et al. 1990, Noguchi et al. 1999)) reveals that they *do* exhibit changes in the above time range. Even in the absence of quinone redox-state changes these might relate to acceptor side processes, e.g., reflecting ionic or proteinaceous relaxation processes following the formation Q_A_^−^. For example, the negative Q_A_^−^ charge may cause subtle rearrangement of the protein-water network at the PSII acceptor side, possibly involving proton relocations, that stabilize Q_A_^−^ and/or are conducive regarding the subsequent Q_B_ reduction. It indeed has been reported that Q_A_ reduction induces changes at the non-heme Fe site, which however were found to be comparatively slow with a half-time of ca. 150 µs (Chernev et al. 2011). The possibility of acceptor side contributions to the IR signals in the time domain from 500 ns to 10 µs is discussed in the following.

At 1658 cm^−1^ the initial IR absorption changes are especially pronounced (see Fig. 2A). This could be due to either P680^+^ or Q_A_^−^, which both absorb at this wavenumber. The flash-number dependency of the 1658 cm^−1^ absorption change between 500 ns and 10 µs, however, does not exhibit a period-of-two pattern (Fig. S11A). A period-of-two pattern would strongly support the contribution of an acceptor side process, whereas a period-of-four pattern is indicative of donor side processes.

Q_A_^−^, P680^+^, and Y_Z_^ox^ all absorb at 1638 cm^−1^ (Noguchi et al. 1999; Nagao et al. 2017), meaning we expect an initially positive IR difference signal, which decays concomitantly with decreasing Q_A_^−^ and P680^+^ populations, but increases as Y_Z_^ox^ is formed. Between 500 ns and 10 µs we indeed observe a rise of the already positive signal before it subsequently decays (not shown); the flash-number dependency of the double-difference absorption again does not show a period-of-two, but a period-of-four behavior (Fig. S11B).

Q_A_^−^, P680^+^ and Y_Z_^ox^ all also have reported positive bands around 1532 cm^−1^ (Noguchi et al. 1999; Nagao et al. 2017). At this wavenumber the double-difference flash dependency might exhibit a small-amplitude period-of-two behavior on top of a larger period-of-four pattern (Fig. S11D).

In conclusion, at four wavenumbers previously assigned to quinones the flash-pattern analysis of Fig. S11 does not provide positive evidence for acceptor-side contributions to the IR signals recorded in the time range from 500 ns to 10 µs. However, this finding does not exclude acceptor side contributions induced by Q_A_^−^ formation (which might then also mask spectral features related to P680^+^ reduction or Y_Z_ oxidation). This is the case because relaxation processes that are induced by Q_A_ oxidation but proceed fully independently of both the Q_B_ site and the non-heme Fe site are not predicted to exhibit a period-of-two flash number dependency. (In our study, the non-heme Fe site undergoes flash-number dependent oxidation state changes in some PSII due to the use of PpBQ as an electron acceptor; see SI for more details.) Thus, there remains the intriguing option that IR transients may report on acceptor side processes trigged by Q_A_ oxidation. Future investigations along this road will be of interest.

### P680^+^ reduction kinetics observed via the broad mid-IR electronic band

By tracking time-resolved IR absorption changes at wavenumbers around 1800 cm^−1^, which is outside the region of protein vibrations, we exploit the broad electronic P680^+^ band (spanning roughly 1000–6000 cm^−1^) (Okubo et al. 2007) to directly observe P680^+^ reduction kinetics. The average of all transients between 1760 and 1884 cm^−1^ (Fig. 3) displays multi-phasic S-state dependent behavior in overall excellent agreement with other spectroscopic studies (Christen et al. 1998; Schilstra et al. 1998). Previously, the P680^+^ decay kinetics were investigated using optical spectroscopy in the visible or near-infrared regime. Using visible-light absorption changes, the probing light also excites the PSII photochemistry; for near-IR probing light (around 820 nm), the time-dependent delayed fluorescence emission (Buchta et al. 2007; Grabolle and Dau 2007; Zaharieva et al. 2011) is sensed by the detector. While work-arounds exist, the absence of these issues when employing time-resolved IR spectroscopy can be seen as a major advantage.

This multi-phasic P680^+^ recombination process has previously been interpreted as arising from conformational changes, possibly involving relocation of protons or water molecules, that shift the P680^+^ Y_Z_ ↔ P680 Y_Z_^ox^ equilibrium more towards the right side of the equation. For example, on the second flash, the fastest phase of P680^+^ reduction and Y_Z_^ox^ formation (about 40 ns) accounts for only about 55% of the total amplitude of the P680^+^ signal (Fig. 8). A value of 50% would correspond to equilibrium constant of unity and thus zero difference of the Gibbs free energy (ΔG_0_) of the reaction. In a subsequent process with a time constant of about 750 ns, the P680^+^ population decreases significantly, corresponding to a negative change in the free-energy difference. The simulation results of Fig. 8 suggest minimally two further processes of decreasing P680^+^ populations, each corresponding to an increasingly negative ΔG_0_.

The 40 ns phase is thought to be linked to, or preceded by, a proton-shift between His190 and Y_Z_ (Eckert and Renger 1988); the application of Marcus theory suggested that it is kinetically limited by a non-adiabatic ET process (Renger et al. 1998). One concept that could reconcile a rate-limiting electron transfer step with a proton transfer is that of a low barrier hydrogen bond (LBHB) between the His190 (of the D1 protein) and Y_Z_ (Renger 2004). Structural analysis of PSII indeed revealed that the distance of the groups in question is shorter than that of a standard H-bond (Saito et al. 2011; Kawashima et al. 2018; Ibrahim et al. 2020; Bhowmick et al. 2023).

The slow nanosecond phase and the microsecond kinetics have been assigned to local (dielectric) and large-scale (proton) relaxation processes, respectively, that further increase the probability of P680^+^ reduction by Y_Z_ (Renger 2004). These steps were also interpreted to have a dual role to (i) stabilize Y_Z_^ox^ and thus reduce the likelihood of charge recombination and (ii) structural changes—incl. restructuring of the protein-water network around Y_Z_ and the Mn_4_CaO_x_ cluster—that prepare the OEC for the subsequent reactions (Klauss et al. 2012b). Klauss et al. also showed that the slow nanosecond events on the second flash and, to a lower extent, on the third flash are accompanied by a volume change of the protein, underlining the idea that structural changes are involved and that these differ between the S-state transitions.

### The broad P680^+^ absorption and its implications for future time-resolved IR studies

The broad electronic P680^+^ band provides the unique opportunity to use mid-IR spectroscopy to trace P680^+^ formation and decay in a wavenumber window (here 1760–1900 cm^−1^) that in PSII is essentially free of distinct IR absorption lines originating from molecular vibrations. Thus, the P680^+^ signal here obtained by detection of IR absorption changes in the range from 1760 to 1900 cm^−1^ is predicted to be free of overlapping contributions from other redox factors or further events (e.g. changing H-bond patters). Such overlap of vibration bands is typically observed in other spectral regions (at wavenumber below 1760 cm^−1^ or well above 1900 cm^−1^), as the extended discussion of the spectra of Fig. 2 vividly illustrates. The broad, featureless P680^+^ background signal has been exploited before for tracking the P680 redox state by detection of IR transients at 4000 cm^−1^ (Sakamoto et al. 2017). Whereas distinct IR absorption lines are not expected to contribute to the broad P680^+^ absorption, further similarly broad absorption changes might contribute, as discussed in the following.

Protonated water clusters or strong hydrogen bonds can exhibit continuum bands that could potentially superimpose the broad P680^+^ band (Zundel 1988). Such a strong H-bond is present between Y_Z_ and D1-His190; H-bonding partners and H-bond strength are changing upon Y_Z_ oxidation or reduction. However, the transients detected at 1884–1760 cm^−1^ do not show any temporal kinetics assignable to Y_z_^ox^ reduction by the Mn_4_CaO_x_ cluster. Thus, we can exclude any significant contribution of the Y_Z_-H-His190 continuum band to the P680^+^ signal (see Figs. 3 and S10). This does not mean that this and other continuum bands are non-existent, but their amplitude most likely is clearly smaller than that of the electronic P680^+^ band. (The signal changes of small amplitude visible in Fig. S10 might relate to such continuum band contributions.)

Broad absorption bands associated with Q_A_ and Q_B_ have been reported between 3000 and 2500 cm^−1^ (Suzuki et al. 2005), and it is conceivable that these also extend also to the wavenumber range studied here. Indeed, the positive signal in the millisecond region of the IR transients recorded at wavenumber above 1760 cm^−1^ (Fig. 3) coincides with the presence of Q_A_^−^ and Q_B_^−^; both quinones seem to contribute roughly equally (Figs. S9 and S10). We estimate their contribution to the initial P680^+^ signal (for a detection system with high time resolution) of being around 5.5% for the first flash applied to dark-adapted PSII and around 3.5% for later flashes (Fig. S10), explaining the y_0_ contribution in Fig. 8 as resulting from the quinone contribution (versus residual P680^+^). We conclude that broad quinone-associated absorption most likely contribute to the IR background signal here assigned to P680^+^. However, its contribution is small and likely fully negligible when discussing the P680^+^ reduction in the time domain from nanoseconds to tens of microsecond.

What further factors influence the broad P680^+^ absorption band and its temporal kinetics? The broad electronic band of PSII samples may be species-dependent or may be influenced by the type of PSII preparation. Okubo et al. (2007) found that PSII membranes from spinach and core complexes of *T. vestitus* exhibit a similar, but not identically broad band; also the broad electronic P700^+^ absorption of PSI particles shows a strong species dependency (Hastings et al. 2001). P680^+^ kinetics differ pronouncedly between intact and Mn-depleted PSII (Haumann et al. 1997; Ahlbrink et al. 1998; Hays et al. 1999). Thus, differing ratios of intact PSII vs. O_2_-inactive PSII is expected to also impact the detected P680^+^ reduction kinetics strongly and thus the background IR signal. Experiments on often more fragile PSII core complexes with genetically modified PSII proteins may be especially strongly affected by slower P680^+^ recombination events due an especially high fraction of O_2_-inactive PSII.

The benefits of detecting P680^+^ transients specifically in the IR regime should not obscure a possible complication relating to the broad electronic transition. Because the electronic band stretches across the entire mid-IR region, it is expected to cause a major time-dependent P680^+^ background signal at all wavenumber in the mid-IR region. In future investigations, it may be crucial to correct for the time-dependent P680^+^ background, because it could affect the time courses assigned to molecular vibrations significantly. An approximate correction can readily be achieved by subtraction of the P680^+^ transients determined at wavenumbers around 1800 cm^−1^. This approach would be accurate for a wavenumber-independent strength of the electronic P680^+^ absorption, but approximative only for a curved P680^+^ absorption background. However, the exact wavenumber dependence of the broad P680^+^ absorption is not known a priori and not readily estimated. One approach for its determination could be to globally fit the time-resolved data set over the entire spectral range between 1890 and 1310 cm^−1^ with a sum of exponential functions, where as an additional summand the P680^+^ transient determined at wavenumbers above 1760 cm^−1^ is weighted with an amplitude that varies only smoothly over the entire wavenumber range, e.g., in form of a quadratic function. We note that in the previously reported step-scan FTIR experiment on oxygen-evolving PSII (Greife et al. 2023), the mode of processing the step-scan data ensured an efficient baseline correction so that the broad electronic contributions assignable to P680^+^ did not affect the time-resolved spectral data set significantly.

In summary, we establish an experimental approach for determination of P680^+^ reduction kinetics in the time range from nanosecond to tens of milliseconds, which is based on detection of the broad electronic absorption band assignable to P680^+^. This absorption band could result in misleading time-resolved data on molecular vibrations, which can be avoided by appropriate corrections; a correction mode is proposed.

### Experimental progress and future developments

Here we demonstrate mid-IR experiments on the light-driven reactions of oxygen-evolving PSII that cover an extended spectral range (1310–1890 cm^−1^) at high time resolution. The methodology is experimentally clearly more efficient than the step-scan FTIR experiment reported in Greife et al (2023).

The fast nanosecond component of 30–50 ns is at the limit of the temporal resolution of the current experimental setup. The here used approach of convolution of the ideal multi-exponential fit function with the IRF nonetheless provides reasonable results but is prone to significant imprecision in the fit parameters. Further improvement of the time resolution could lead to an increased robustness of the quantitative results (regarding values of time constants and amplitudes in multi-exponential simulations).

In summary, we believe that the present study paves the way for future time-resolved IR experiments on PSII at high spectral and temporal resolution. Further technical developments are conceivable that improve the performance of the experiment further.

## Supplementary Information

Below is the link to the electronic supplementary material.

Supplementary file1 (PDF 1742 KB)

## Data Availability

All data that is relevant for our conclusions is shown in the article or the Supporting Information file.

## References

[CR1] Ahlbrink R, Haumann M, Cherepanov D, Boegershausen O, Mulkidjanian A, Junge W (1998) Function of tyrosine-Z in water oxidation by photosystem II: electrostatical promotor instead of hydrogen abstractor. Biochemistry 37:1131–1142. 10.1021/bi9719152 9454606 10.1021/bi9719152

[CR2] Babcock GT, Sauer K (1975) A rapid, light-induced transient in electron paramagnetic resonance signal II activated upon inhibition of photosynthetic oxygen evolution. Biochim Biophys Acta 376:315–328. 10.1016/0005-2728(75)90024-9 163649 10.1016/0005-2728(75)90024-9

[CR3] Berthold DA, Babcock GT, Yocum CF (1981) A highly resolved, oxygen-evolving photosystem-II preparation from spinach thylakoid membranes—EPR and electron-transport properties. FEBS Lett 134:231–234. 10.1016/0014-5793(81)80608-4

[CR4] Berthomieu C, Hienerwadel R (2005) Vibrational spectroscopy to study the properties of redox-active tyrosines in photosystem II and other proteins. Biochim Biophys Acta 1707:51–66. 10.1016/j.bbabio.2004.03.011 15721606 10.1016/j.bbabio.2004.03.011

[CR5] Berthomieu C, Nabedryk E, Mantele W, Breton J (1990) Characterization by FTIR spectroscopy of the photoreduction of the primary quinone acceptor Q_A_ in photosystem II. FEBS Lett 269:363–367. 10.1016/0014-5793(90)81194-S 15452972 10.1016/0014-5793(90)81194-s

[CR6] Berthomieu C, Hienerwadel R, Boussac A, Breton J, Diner BA (1998) Hydrogen bonding of redox-active tyrosine Z of photosystem II probed by FTIR difference spectroscopy. Biochemistry 37:10547–10554. 10.1021/bi980788m 9692943 10.1021/bi980788m

[CR7] Bhowmick A, Hussein R, Bogacz I, Simon PS, Ibrahim M, Chatterjee R, Doyle MD, Cheah MH, Fransson T, Chernev P, Kim I-S, Makita H, Dasgupta M, Kaminsky CJ, Zhang M, Gätcke J, Haupt S, Nangca II, Keable SM, Aydin AO, Tono K, Owada S, Gee LB, Fuller FD, Batyuk A, Alonso-Mori R, Holton JM, Paley DW, Moriarty NW, Mamedov F, Adams PD, Brewster AS, Dobbek H, Sauter NK, Bergmann U, Zouni A, Messinger J, Kern J, Yano J, Yachandra VK (2023) Structural evidence for intermediates during O_2_ formation in photosystem II. Nature 617:629–636. 10.1038/s41586-023-06038-z 37138085 10.1038/s41586-023-06038-z PMC10191843

[CR8] Breton J, Nabedryk E, Parson WW (1992) A new infrared electronic transition of the oxidized primary electron donor in bacterial reaction centers: a way to assess resonance interactions between the bacteriochlorophylls. Biochemistry 31:7503–7510. 10.1021/bi00148a010 1510937 10.1021/bi00148a010

[CR9] Breton J, Nabedryk E, Leibl W (1999) FTIR study of the primary electron donor of photosystem I (P700) revealing delocalization of the charge in P700^+^ and localization of the triplet character in ^3^P700. Biochemistry 38:11585–11592. 10.1021/bi991216k 10512612 10.1021/bi991216k

[CR10] Brettel K, Schlodder E, Witt HT (1984) Nanosecond reduction kinetics of photooxidized chlorophyll-*a*_II_ (P-680) in single flashes as a probe for the electron pathway, H^+^-release and charge accumulation in the O_2_-evolving complex. Biochim Biophys Acta 766:403–415. 10.1016/0005-2728(84)90256-1

[CR11] Buchta J, Grabolle M, Dau H (2007) Photosynthetic dioxygen formation studied by time-resolved delayed fluorescence measurements—method, rationale, and results on the activation energy of dioxygen formation. Biochim Biophys Acta 1767:565–574. 10.1016/j.bbabio.2007.04.003 17543884 10.1016/j.bbabio.2007.04.003

[CR12] Chernev P, Zaharieva I, Dau H, Haumann M (2011) Carboxylate shifts steer inter-quinone electron transfer in photosynthesis. J Biol Chem 286:5368–5374. 10.1074/jbc.M110.202879 21169354 10.1074/jbc.M110.202879 PMC3037649

[CR13] Christen G, Renger G (1999) The role of hydrogen bonds for the multiphasic P680^+•^ reduction by Y_Z_ in photosystem II with intact oxygen evolution capacity. Analysis of kinetic H/D isotope exchange effects. Biochemistry 38:2068–2077. 10.1021/bi982188t 10026289 10.1021/bi982188t

[CR14] Christen G, Reifarth F, Renger G (1998) On the origin of the ‘35-μs kinetics’ of P680^+•^ reduction in photosystem II with an intact water oxidising complex. FEBS Lett 429:49–52. 10.1016/S0014-5793(98)00552-3 9657382 10.1016/s0014-5793(98)00552-3

[CR15] Christen G, Seeliger A, Renger G (1999) P680^+•^ reduction kinetics and redox transition probability of the water oxidizing complex as a function of pH and H/D isotope exchange in spinach thylakoids. Biochemistry 38:6082–6092. 10.1021/bi9827520 10320334 10.1021/bi9827520

[CR16] Cox N, Pantazis DA, Neese F, Lubitz W (2015) Artificial photosynthesis: understanding water splitting in nature. Interface Focus 5:20150009. 10.1098/rsfs.2015.0009 26052426 10.1098/rsfs.2015.0009 PMC4410565

[CR17] Cox N, Pantazis DA, Lubitz W (2020) Current understanding of the mechanism of water oxidation in photosystem II and its relation to XFEL data. Annu Rev Biochem 89:795–820. 10.1146/annurev-biochem-011520-104801 32208765 10.1146/annurev-biochem-011520-104801

[CR18] Dau H, Haumann M (2007) Eight steps preceding O–O bond formation in oxygenic photosynthesis—a basic reaction cycle of the photosystem II manganese complex. Biochim Biophys Acta 1767:472–483. 10.1016/j.bbabio.2007.02.022 17442260 10.1016/j.bbabio.2007.02.022

[CR19] Dau H, Zaharieva I (2009) Principles, efficiency, and blueprint character of solar-energy conversion in photosynthetic water oxidation. Acc Chem Res 42:1861–1870. 10.1021/ar900225y 19908828 10.1021/ar900225y

[CR20] Dau H, Limberg C, Reier T, Risch M, Roggan S, Strasser P (2010) The mechanism of water oxidation: from electrolysis via homogeneous to biological catalysis. ChemCatChem 2:724–761. 10.1002/cctc.201000126

[CR21] Debus RJ (2015) FTIR studies of metal ligands, networks of hydrogen bonds, and water molecules near the active site Mn_4_CaO_5_ cluster in photosystem II. Biochim Biophys Acta 1847:19–34. 10.1016/j.bbabio.2014.07.007 25038513 10.1016/j.bbabio.2014.07.007

[CR22] Eckert HJ, Renger G (1988) Temperature dependence of P680^+^ reduction in O_2_-evolving PS II membrane fragments at different redox states S_i_ of the water oxidizing system. FEBS Lett 236:425–431. 10.1016/0014-5793(88)80070-X

[CR23] Eckert HJ, Wiese N, Bernarding J, Eichler HJ, Renger G (1988) Analysis of the electron transfer from Pheo^−^ to Q_A_ in PS II membrane fragments from spinach by time resolved 325 nm absorption changes in the picosecond domain. FEBS Lett 240:153–158. 10.1016/0014-5793(88)80358-2 3056745 10.1016/0014-5793(88)80358-2

[CR24] Gläser M, Wolff C, Renger G (1976) Indirect evidence for a very fast recovery kinetics of chlorophyll-a_II_ in spinach chloroplasts. Zeitschrift Für Naturforschung C 31:712–721. 10.1515/znc-1976-11-1216 10.1515/znc-1976-11-1216 138292

[CR25] Grabolle M, Dau H (2007) Efficiency and role of loss processes in light-driven water oxidation by PSII. Physiol Plantarum 131:50–63. 10.1111/j.1399-3054.2007.00941.x 10.1111/j.1399-3054.2007.00941.x 18251924

[CR26] Greife P, Schönborn M, Capone M, Assunção R, Narzi D, Guidoni L, Dau H (2023) The electron–proton bottleneck of photosynthetic oxygen evolution. Nature 617:623–628. 10.1038/s41586-023-06008-5 37138082 10.1038/s41586-023-06008-5 PMC10191853

[CR27] Hastings G, Ramesh V, Wang R, Sivakumar V, Webber A (2001) Primary donor photo-oxidation in photosystem I: a re-evaluation of (P700^+^− P700) Fourier transform infrared difference spectra. Biochemistry 40:12943–12949. 10.1021/bi0155753 11669631 10.1021/bi0155753

[CR28] Haumann M, Bögershausen O, Cherepanov D, Ahlbrink R, Junge W (1997) Photosynthetic oxygen evolution: H/D isotope effects and the coupling between electron and proton transfer during the redox reactions at the oxidizing side of photosystem II. Photosynth Res 51:193–208. 10.1023/A:1005861917596

[CR29] Hays AM, Vassiliev IR, Golbeck JH, Debus RJ (1999) Role of D1-His190 in the proton-coupled oxidation of tyrosine Y_Z_ in manganese-depleted photosystem II. Biochemistry 38:11851–11865. 10.1021/bi990716a 10508388 10.1021/bi990716a

[CR30] Hienerwadel R, Boussac A, Breton J, Berthomieu C (1996) Fourier transform infrared difference study of tyrosine_D_ oxidation and plastoquinone Q_A_ reduction in photosystem II. Biochemistry 35:15447–15460. 10.1021/bi961952d 8952498 10.1021/bi961952d

[CR31] Ibrahim M, Fransson T, Chatterjee R, Cheah MH, Hussein R, Lassalle L, Sutherlin KD, Young ID, Fuller FD, Gul S, Kimc I-S, Simon PS, Lichtenberg Cd, Chernev P, Bogacz I, Pham CC, Orville AM, Saichek N, Northen T, Batyuk A, Carbajo S, Alonso-Mori R, Tono K, Owada S, Bhowmick A, Bolotovsky R, Mendez D, Moriarty NW, Holton JM, Dobbek H, Brewster AS, Adams PD, Sauter NK, Bergmann U, Zouni A, Messinger J, Kern J, Yano VKYaJ (2020) Untangling the sequence of events during the S_2_→ S_3_ transition in photosystem II and implications for the water oxidation mechanism. Proc Natl Acad Sci USA 117:12624–12635. 10.1073/pnas.200052911 32434915 10.1073/pnas.2000529117 PMC7293653

[CR32] Jeans C, Schilstra MJ, Klug DR (2002) The temperature dependence of P680^+^ reduction in oxygen-evolving photosystem II. Biochemistry 41:5015–5023. 10.1021/bi0118862 11939798 10.1021/bi0118862

[CR33] Junge W (2019) Oxygenic photosynthesis: history, status and perspective. Q Rev Biol 52:E1. 10.1017/S0033583518000112 10.1017/S0033583518000112 30670110

[CR34] Karge M, Irrgang KD, Sellin S, Feinaugle R, Liu B, Eckert HJ, Eichler HJ, Renger G (1996) Effects of hydrogen/deuterium exchange on photosynthetic water cleavage in PS II core complexes from spinach. FEBS Lett 378:140–144. 10.1016/0014-5793(95)01433-0 8549820 10.1016/0014-5793(95)01433-0

[CR35] Kato Y, Ishii R, Noguchi T (2016) Comparative analysis of the interaction of the primary quinone Q_A_ in intact and Mn-depleted photosystem II membranes using light-induced ATR-FTIR spectroscopy. Biochemistry 55:6355–6358. 10.1021/acs.biochem.6b01052 27933774 10.1021/acs.biochem.6b01052

[CR36] Kawashima K, Saito K, Ishikita H (2018) Mechanism of radical formation in the H-bond network of D1-Asn298 in photosystem II. Biochemistry 57:4997–5004. 10.1021/acs.biochem.8b00574 30015472 10.1021/acs.biochem.8b00574

[CR37] Klauss A, Haumann M, Dau H (2012a) Alternating electron and proton transfer steps in photosynthetic water oxidation. Proc Natl Acad Sci USA 109:16035–16040. 10.1073/pnas.1206266109 22988080 10.1073/pnas.1206266109 PMC3479599

[CR38] Klauss A, Sikora T, Suss B, Dau H (2012b) Fast structural changes (200–900ns) may prepare the photosynthetic manganese complex for oxidation by the adjacent tyrosine radical. Biochim Biophys Acta 1817:1196–1207. 10.1016/j.bbabio.2012.04.017 22579714 10.1016/j.bbabio.2012.04.017

[CR39] Kok B, Forbush B, McGloin M (1970) Cooperation of charges in photosynthetic O_2_ evolution-I. A linear four-step mechanism. Photochem Photobiol 11:457–475. 10.1111/j.1751-1097.1970.tb06017.x 5456273 10.1111/j.1751-1097.1970.tb06017.x

[CR40] Kühn P, Eckert H-J, Eichler H, Renger G (2004) Analysis of the P680^+^˙ reduction pattern and its temperature dependence in oxygen-evolving PS II core complexes from thermophilic cyanobacteria and higher plants. Phys Chem Chem Phys 6:4838–4843. 10.1039/B407656G

[CR41] Lubitz W, Chrysina M, Cox N (2019) Water oxidation in photosystem II. Photosynth Res 142:105–125. 10.1007/s11120-019-00648-3 31187340 10.1007/s11120-019-00648-3 PMC6763417

[CR42] Lukins PB, Post A, Walker PJ, Larkum AW (1996) P680^+^ reduction in oxygen-evolving photosystem II core complexes. Photosynth Res 49:209–221. 10.1007/BF00034782 24271699 10.1007/BF00034782

[CR43] Mäusle SM, Abzaliyeva A, Greife P, Simon PS, Perez R, Zilliges Y, Dau H (2020) Activation energies for two steps in the S_2_ → S_3_ transition of photosynthetic water oxidation from time-resolved single-frequency infrared spectroscopy. J Chem Phys 153:215101. 10.1063/5.0027995 33291916 10.1063/5.0027995

[CR44] Mäusle SM, Agarwala N, Eichmann VG, Dau H, Nürnberg DJ, Hastings G (2023) Nanosecond time-resolved infrared spectroscopy for the study of electron transfer in photosystem I. Photosynth Res. 10.1007/s11120-023-01035-9 37420121 10.1007/s11120-023-01035-9 PMC10991071

[CR45] Meyer B, Schlodder E, Dekker JP, Witt HT (1989) O_2_ evolution and Chl *a*^+II^ (P-680^+^) nanosecond reduction kinetics in single flashes as a function of pH. Biochim Biophys Acta 974:36–43. 10.1016/S0005-2728(89)80163-X

[CR46] Nagao R, Yamaguchi M, Nakamura S, Ueoka-Nakanishi H, Noguchi T (2017) Genetically introduced hydrogen bond interactions reveal an asymmetric charge distribution on the radical cation of the special-pair chlorophyll P680. J Biol Chem 292:7474–7486. 10.1074/jbc.M117.781062 28302724 10.1074/jbc.M117.781062 PMC5418047

[CR47] Nakamura S, Nagao R, Takahashi R, Noguchi T (2014) Fourier transform infrared detection of a polarizable proton trapped between photooxidized tyrosine Y_Z_ and a coupled histidine in photosystem II: relevance to the proton transfer mechanism of water oxidation. Biochemistry 53:3131–3144. 10.1021/bi500237y 24786306 10.1021/bi500237y

[CR48] Noguchi T (2010) Fourier transform infrared spectroscopy of special pair bacteriochlorophylls in homodimeric reaction centers of heliobacteria and green sulfur bacteria. Photosynth Res 104:321–331. 10.1007/s11120-009-9509-0 20094792 10.1007/s11120-009-9509-0

[CR49] Noguchi T (2015) Fourier transform infrared difference and time-resolved infrared detection of the electron and proton transfer dynamics in photosynthetic water oxidation. Biochim Biophys Acta 1847:35–45. 10.1016/j.bbabio.2014.06.009 24998309 10.1016/j.bbabio.2014.06.009

[CR50] Noguchi T, Tomo T, Inoue Y (1998) Fourier transform infrared study of the cation radical of P680 in the photosystem II reaction center: evidence for charge delocalization on the chlorophyll dimer. Biochemistry 37:13614–13625. 10.1021/bi9812975 9753448 10.1021/bi9812975

[CR51] Noguchi T, Inoue Y, Tang X-S (1999) Hydrogen bonding interaction between the primary quinone acceptor Q_A_ and a histidine side chain in photosystem II as revealed by Fourier transform infrared spectroscopy. Biochemistry 38:399–403. 10.1021/bi982294v 9890922 10.1021/bi982294v

[CR52] Noguchi T, Suzuki H, Tsuno M, Sugiura M, Kato C (2012) Time-resolved infrared detection of the proton and protein dynamics during photosynthetic oxygen evolution. Biochemistry 51:3205–3214. 10.1021/bi300294n 22458839 10.1021/bi300294n

[CR53] Nuijs AM, Vangorkom HJ, Plijter JJ, Duysens LNM (1986) Primary-charge separation and excitation of chlorophyll *a* in photosystem II particles from spinach as studied by picosecond absorbance-difference spectroscopy. Biochim Biophys Acta 848:167–175. 10.1016/0005-2728(86)90038-1

[CR54] Okubo T, Tomo T, Sugiura M, Noguchi T (2007) Perturbation of the structure of P680 and the charge distribution on its radical cation in isolated reaction center complexes of photosystem II as revealed by Fourier transform infrared spectroscopy. Biochemistry 46:4390–4397. 10.1021/bi700157n 17371054 10.1021/bi700157n

[CR55] Renger G (2004) Coupling of electron and proton transfer in oxidative water cleavage in photosynthesis. Biochim Biophys Acta 1655:195–204. 10.1016/j.bbabio.2003.07.007 15100032 10.1016/j.bbabio.2003.07.007

[CR56] Renger G, Christen G, Karge M, Eckert H-J, Irrgang K-D (1998) Application of the Marcus theory for analysis of the temperature dependence of the reactions leading to photosynthetic water oxidation: results and implications. J Biol Inorg Chem 3:360–366. 10.1007/s007750050245

[CR57] Saito K, Shen J-R, Ishida T, Ishikita H (2011) Short hydrogen bond between redox-active tyrosine Y_Z_ and D1-His190 in the photosystem II crystal structure. Biochemistry 50:9836–9844. 10.1021/bi201366j 21972783 10.1021/bi201366j

[CR58] Sakamoto H, Shimizu T, Nagao R, Noguchi T (2017) Monitoring the reaction process during the S_2_→S_3_ transition in photosynthetic water oxidation using time-resolved infrared spectroscopy. J Am Chem Soc 139:2022–2029. 10.1021/jacs.6b11989 28088851 10.1021/jacs.6b11989

[CR59] Schiller H, Dau H (2000) Preparation protocols for high-activity photosystem II membrane particles of green algae and higher plants, pH dependence of oxygen evolution and comparison of the S_2_-state multiline signal by X-band EPR spectroscopy. J Photochem Photobiol B 55:138–144. 10.1016/S1011-1344(00)00036-1 10942078 10.1016/s1011-1344(00)00036-1

[CR60] Schilstra MJ, Rappaport F, Nugent JHA, Barnett CJ, Klug DR (1998) Proton/hydrogen transfer affects the S-state-dependent microsecond phases of P680^+^ reduction during water splitting. Biochemistry 37:3974–3981. 10.1021/bi9713815 9521719 10.1021/bi9713815

[CR61] Schlodder E, Brettel K, Witt HT (1985) Relation between microsecond reduction kinetics of photooxidized chlorophyll *a*_II_ (P-680) and photosynthetic water oxidation. Biochim Biophys Acta 808:123–131. 10.1016/0005-2728(85)90034-9

[CR62] Shevela D, Kern JF, Govindjee G, Messinger J (2023) Solar energy conversion by photosystem II: principles and structures. Photosynth Res 156:79–307. 10.1007/s11120-022-00991-y 10.1007/s11120-022-00991-y PMC10203033 36826741

[CR63] Styring S, Sjöholm J, Mamedov F (2012) Two tyrosines that changed the world: Interfacing the oxidizing power of photochemistry to water splitting in photosystem II. Biochim Biophys Acta 1817:76–87. 10.1016/j.bbabio.2011.03.016 21557928 10.1016/j.bbabio.2011.03.016

[CR64] Suzuki H, Nagasaka M-a, Sugiura M, Noguchi T (2005) Fourier transform infrared spectrum of the secondary quinone electron acceptor Q_B_ in photosystem II. Biochemistry 44:11323–11328. 10.1021/bi051237g 16114869 10.1021/bi051237g

[CR65] Takahashi R, Hasegawa K, Noguchi T (2008) Effect of charge distribution over a chlorophyll dimer on the redox potential of P680 in photosystem II as studied by density functional theory calculations. Biochemistry 47:6289–6291. 10.1021/bi8007998 18500822 10.1021/bi8007998

[CR66] Takemoto H, Sugiura M, Noguchi T (2019) Proton release process during the S_2_-to-S_3_ transition of photosynthetic water oxidation as revealed by the pH dependence of kinetics monitored by time-resolved infrared spectroscopy. Biochemistry 58:4276–4283. 10.1021/acs.biochem.9b00680 31568726 10.1021/acs.biochem.9b00680

[CR67] Umena Y, Kawakami K, Shen J-R, Kamiya N (2011) Crystal structure of oxygen-evolving photosystem II at a resolution of 1.9 Å. Nature 473:55–60. 10.1038/nature09913 21499260 10.1038/nature09913

[CR68] Vinyard DJ, Brudvig GW (2017) Progress toward a molecular mechanism of water oxidation in photosystem II. Annu Rev Phys Chem 68(68):101–116. 10.1146/annurev-physchem-052516-044820 28226223 10.1146/annurev-physchem-052516-044820

[CR69] Zaharieva I, Wichmann JM, Dau H (2011) Thermodynamic limitations of photosynthetic water oxidation at high proton concentrations. J Biol Chem 286:18222–18228. 10.1074/jbc.M111.237941 21464129 10.1074/jbc.M111.237941 PMC3093894

[CR70] Zhang HM, Razeghifard MR, Fischer G, Wydrzynski T (1997) A time-resolved FTIR difference study of the plastoquinone Q_A_ and redox-active tyrosine Y_Z_ interactions in photosystem II. Biochemistry 36:11762–11768. 10.1021/bi970815t 9305966 10.1021/bi970815t

[CR71] Zundel G (1988) Proton transfer in and proton polarizability of hydrogen bonds: IR and theoretical studies regarding mechanisms in biological systems. J Mol Struct 177:43–68. 10.1016/0022-2860(88)80078-4

