# Supplementary material for: Tracking the first electron transfer step at the donor side of oxygen-evolving photosystem II by time-resolved infrared spectroscopy
Source: Photosynth Res. 2023 Nov 23;162(2-3):353–69. doi: 10.1007/s11120-023-01057-3 (PMC11615052; doi:10.1007/s11120-023-01057-3)
Supplement: Supplementary file 1 — Supplementary file1 (PDF 1742 KB) [file 11120_2023_1057_MOESM1_ESM.pdf]

Supplementary Information

in support of

Tracking the first electron transfer step at the donor side of oxygen-evolving photosystem II by time-resolved infrared spectroscopy

Mohamad Yahia Dekmak[1], Sarah M. Mäusle[1, *], Janosch Brandhorst[1], Philipp S. Simon[1,2], Holger Dau[1, *]

1 Department of Physics, Freie Universität Berlin, Berlin, Germany.

2 Molecular Biophysics and Integrated Bioimaging Division, Lawrence Berkeley National Laboratory, Berkeley, CA, USA.

* Authors to whom correspondence should be addressed:

holger.dau@fu-berlin.de; sarah.maeusle@fu-berlin.de

1. $P680^+$ Transients: Details on the Fitting Procedure and Calculated Fit Parameters

Transients were fit in the range of 0-100 µs to a model defined by a sum of four exponentials parametrized as follows:

$$y(t) = y_o + a_1 e^{-t/\tau_1} + a_2 e^{-t/\tau_2} + a_3 e^{-t/\tau_3} + a_4 e^{-t/\tau_4}. \qquad \text{(Eq. S1)}$$

Additionally, an instrument response function (IRF) was taken into consideration which was iteratively convolved with the multi-exponential model during the least-squares optimization process. This was done to account for the laser pulse width of about 5 ns FWHM (full width at half maximum) and response time of the detector preamplifier (ca. 15 ns corresponding to 10 MHz cut-off frequency). The IRF is approximated as

$$\text{IRF}(\tau) = e^{\frac{-(\tau-\tau_O)^2}{2\sigma^2}}, \qquad \text{(Eq. S2)}$$

with σ = 17 ns and t_0 = 44 ns.

The convolution of the ideal time response of PSII (Eq. S1) and the IRF (Eq. S2) was calculated according to:

$$\Delta A(t) = \int_{-\infty}^{\infty} y\,(t-\tau) IRF(\tau) d\tau. \qquad \text{(Eq. 3)}$$

For determination of the fit parameters in Eq. 1 (fit parameters) by least-squares curve fitting, the error sum was calculated as the difference between values of $\Delta A(t)$ calculated by Eq. 1 and the corresponding experimental values. The error sum was calculated for all data points within the fit range, which was selected to be 1 - 100 µs. The search areas for the time constants and amplitudes were constrained as given in Table S1. Additional constraints were that the sum of all amplitudes and y_0 should be the same for all flashes (i.e., $a_1+a_2+a_3+a_4+y_0$ = const.). The slowest time constant τ_4 was constrained to be the same for all four flashes.

A comprehensive overview of the fitting results is given in Fig S1. The explicit parameters found during the fitting procedure are given in Tables S2 and S3.

Table S1: Boundaries of the fit parameters during least-squares optimization. For every parameter a minimum and maximum value is given, defining an area in which feasible solutions are searched. NA indicates that no limit is defined with respect to the given direction.

	τ_1 (ns)	τ_2 (ns)	τ_3 (µs)	τ_4 (µs)	a_1 (mOD)	a_2 (mOD)	a_3 (mOD)	a_4 (mOD)	y_0 (mOD)
**min**	20	100	2	20	0	0	0	0	-1
**max**	100	2000	12	70	NA	NA	NA	NA	1

Table S2: Amplitudes and offset determined by fitting the flash induced IR transients of wavenumbers greater than 1760 cm^{-1} to a multi-exponential function. The given 1σ error ranges are the standard error as determined from the covariance matrix provided by the least-squares optimization algorithm. The values are the same as shown in Fig. S1.

Flash	a_1(mOD)	a_2(mOD)	a_3(mOD)	a_4(mOD)	y_0(mOD)
**1**	0.618 ± 0.035	0.113 ± 0.011	0.025 ± 0.007	0.043 ± 0.005	0.046 ±0.002
**2**	0.491 ± 0.034	0.202 ± 0.015	0.041 ± 0.013	0.079 ± 0.004	0.032 ± 0.002
**3**	0.512 ± 0.035	0.149 ± 0.015	0.063 ± 0.013	0.089 ± 0.004	0.034 ± 0.002
**4**	0.621± 0.035	0.081 ± 0.010	0.044 ± 0.008	0.065 ± 0.006	0.042 ± 0.004

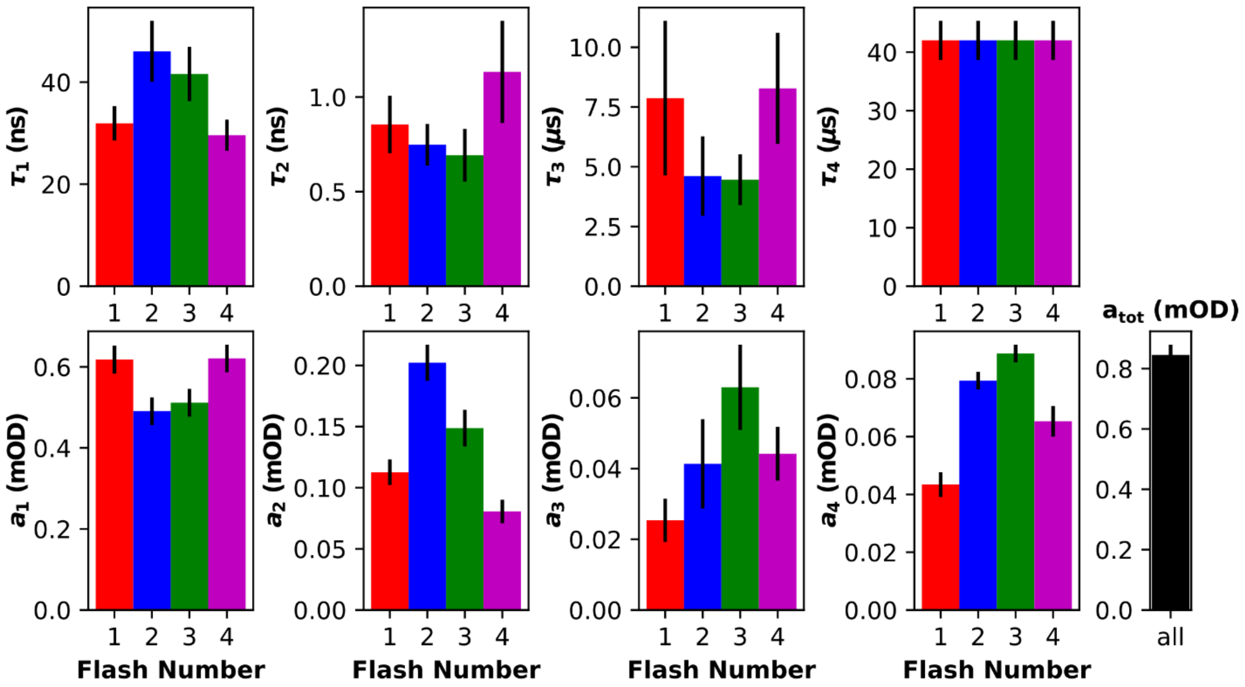

Figure S1: Fit parameters of the multi-exponential fits of the IR transients obtained at wavenumbers greater than 1760 cm^{-1}. The first row indicates the time constants of the four components determined for the multi-exponential function of Eq. S1. The second row indicates the amplitudes of the corresponding components. The black bars indicate the standard error, determined from the covariance matrix provided by the least-squares optimization algorithm. Fitting was done under the constraint that the slowest time constant as well as the sum of all amplitudes plus offset is the same for all flashes. Fit parameters associated with the $S_1 \rightarrow S_2$ transition (1st flash) are shown in red, the $S_2 \rightarrow S_3$ transition (2nd flash) in blue, the $S_3 \rightarrow S_0$ transition (3rd flash) in green, and the $S_0 \rightarrow S_1$ (4th flash) in magenta.

Table S3: Time constants determined by fitting the flash induced IR transients of wavenumbers greater than 1760 cm^{-1} to a multi-exponential function. The given 1σ error ranges are the standard error as determined from the covariance matrix provided by the least-squares optimization algorithm. The values are the same as plotted in Fig. S1.

Flash	τ_1 (ns)	τ_2 (ns)	τ_3 (µs)	τ_4 (µs)
**1**	32.9 ± 3.4	855 ± 152	7.9 ± 3.2	42.0 ± 3.3
**2**	46.0 ± 6.0	747 ± 110	4.6 ± 1.7	42.0 ± 3.3
**3**	41.6 ± 5.3	692 ± 139	4.5 ± 1.1	42.0 ± 3.3
**4**	29.6 ± 3.1	1133 ± 270	8.3 ± 2.3	42.0 ± 3.3

2. P680⁺ Transients: Additional Information on Peak Suppression by IRF

As it is not immediately clear why the peak intensity in the 2nd and 3rd flash are higher than the peak intensity in the 1st and 4th flashes, we convolved a four component multi-exponential model with a previously determined Gaussian IRF (Mäusle et al. 2023). Figure S2 shows the effect of such a convolution process on the measurement of hypothetical two-component multi-exponentials. Figure S3 is very similar to the respective figure presented in the main paper (Fig. 6). However, the presence of multiple exponential components has a qualitatively different effect on the position of the peak of recorded transient with respect to the fast time constant. Importantly the presence of additional components in a multi-exponential model still offers an explanation for the unexpected differences in peak amplitude observed in the different flash induced IR-transients.

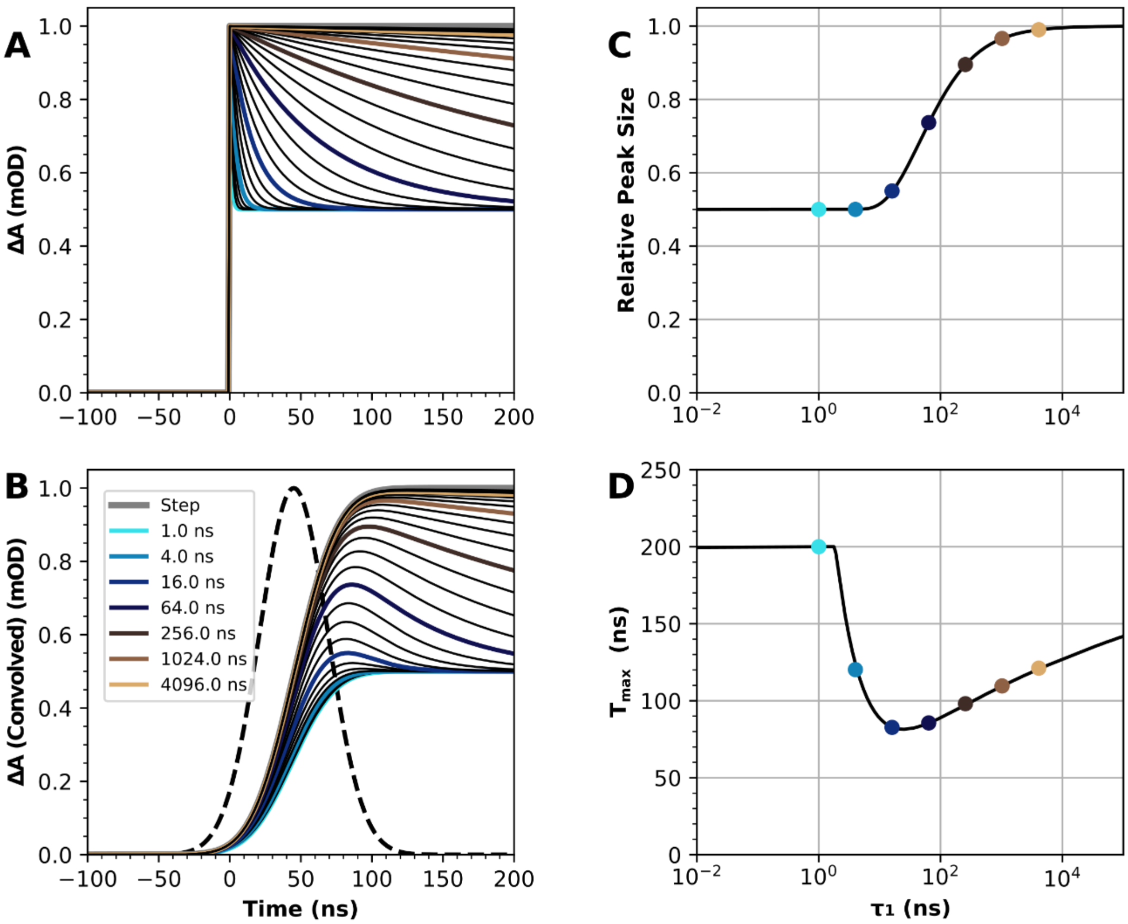

Figure S2: Influence of a Gaussian instrument response function (IRF) on the peak intensity of idealized hypothetical multi-exponential transients composed of a fast component and a very slow component. **(A)** Idealized transients each composed of an infinitely slow component and a very fast component. The fast components range from 1 ns to 4 µs and are indicated by colored lines. Black, thinner lines in between the colored lines indicate fast components with values logarithmically placed in between those of the

colored lines. The thicker gray line indicates a transient with only an infinitely slow time constant as a limit case. **(B)** The effect of convolving the idealized transients from A with the instrument response function indicated by the black dashed line. The convolved transients are shown in the same color code as in A. The data shown in B is the same as shown in Fig. 6E of the main paper. **(C)** Influence of the fast time constant of the exponential function on the peak intensity of the convolved transients. **(D)** Influence on the temporal position of the peak as a function of the fast time constant. The colored dots in C and D correspond to the transients shown in A and B.

3. Alternative Data Visualization – Non-Smoothed Spectra & Transients plus Additional Figures Illustrating Fit Results

Figure S3 refers to the same data as shown in Fig. 2 of the main paper, but here there was no smoothing algorithm applied. Additionally, a dark spectrum (measured without exciting the sample by a laser flash) for all selected time points (80 ns, 500 ns and 10 µs) is shown in gray, representing the noise level.

The baseline correction of the data in Fig. 2E and Fig. S3E was performed by first, subtracting the average of all data values across the entire spectral range individually for the data sets of the 1st, 2nd, 3rd, and 4th flash. Second, the mean value of all four data sets was calculated in the range from 1760 to 1810 cm^{-1} and subtracted.

Figure S4 shows all ten flash-induced transients at wavenumbers greater than 1760 cm^{-1} data (of which the first four are shown in Fig. 3 of the main paper). Compared to all subsequent flashes, the first flash exhibits an up-shifted signal in the range from ~100 µs to 800 ms, while all other flashes look identical in that region, for presently not understood reasons.

IR-transients recorded after the first four flashes were fitted as described before in the range 0 to 100 µs. The transients overlayed with the fit curves for the complete time range can be seen on a logarithmic time axis in Fig S5. The weighted residuals, i.e., the weighted difference between the fit and the recorded data, are also shown. The weighting is done by taking into account that later timepoints represent larger amount of data due to quasi-logarithmic sampling of timepoints. Fig. S6 shows a section of the same data between 1.5 µs before the laser excitation until 10 µs after the excitation on a linear scale; the fit errors are again given by the weighted residuals. Both figures represent the same fits and data as shown in the main paper (Fig. 7).

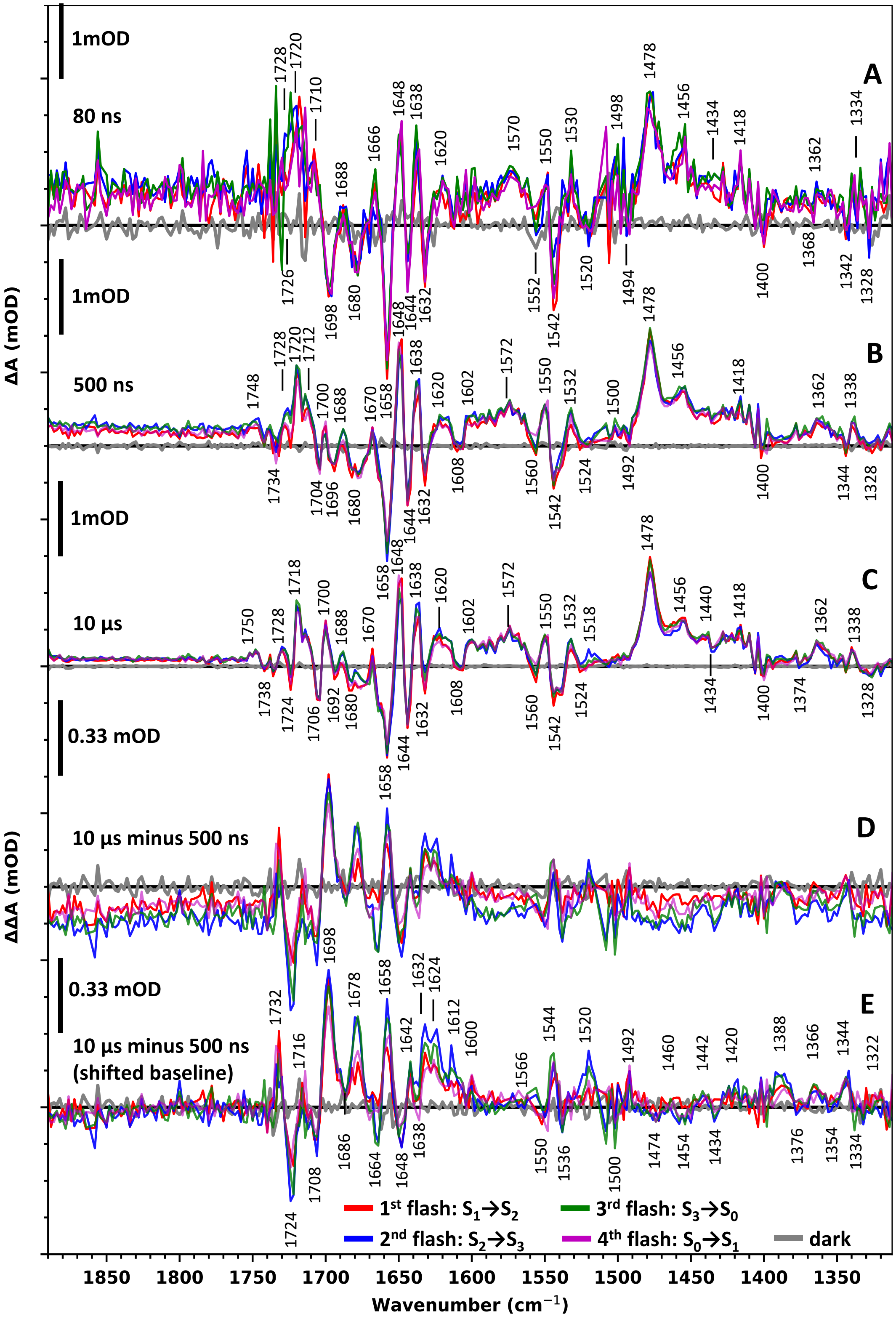

Figure S3: Flash-induced time resolved IR difference spectra of spinach PSII membrane particles at 10°C. The difference absorbance (ΔA) was obtained at every ~2 cm^{-1} between ~1310 and 1890 cm^{-1} by

acquiring flash-induced transients. **(A)** This spectrum was obtained by averaging the transient data acquired between 50 and 110 ns. **(B)** IR difference spectrum of around 500 ns (250-750 ns) after excitation. **(C)** IR difference spectrum of around 10 µs (8-12 µs) after excitation. **(D)** Double difference spectrum of the 10 µs spectrum minus the 500 ns spectrum (C – B). The resulting double difference absorption (ΔΔA) was small compared to the data in A-C and was thus multiplied by a factor of 3 for better visualization. **(E)** A baseline-corrected version of the double difference spectrum shown in D, i.e. the spectrum of 10 µs minus 500 ns. The noise level is indicated by dark spectra (grey), obtained in the same way as the flash-induced data, but without applying excitation flashes

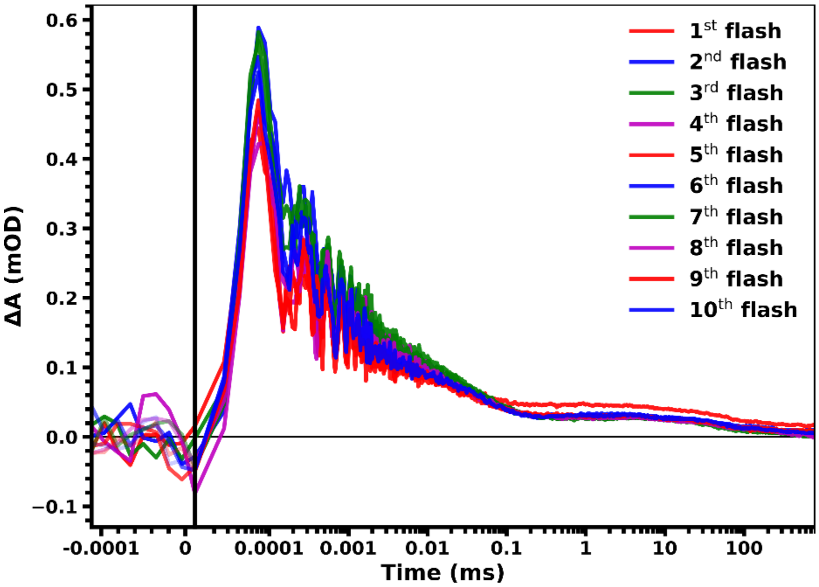

Figure S4: Time-dependence of the broad background in the infrared absorption detected above 1760 cm^{-1}. All time resolved IR-transients acquired between 1760 – 1884 cm^{-1} were averaged to improve the SNR. All flash-induced transients, obtained by a sequence of ten excitation flashes, are shown. Data associated mostly with the $S_1 \rightarrow S_2$ transition (1st, 5th and 9th flash) is shown in red, the $S_2 \rightarrow S_3$ transition (2nd, 6th and 10th flash) in blue, the $S_3 \rightarrow S_0$ transition (3rd and 7th flash) in (green) and the $S_0 \rightarrow S_1$ (4th and 8th flash) in magenta. The signal from 100 ns before the excitation flash up to 11.5 ns after the flash is shown on a linear scale, while the data between 11.5 ns and 800 ms is shown on a logarithmic x-axis; the two different axes are separated by a vertical line. The first four flash-induced transients are also shown in Fig. 3 of the main paper.

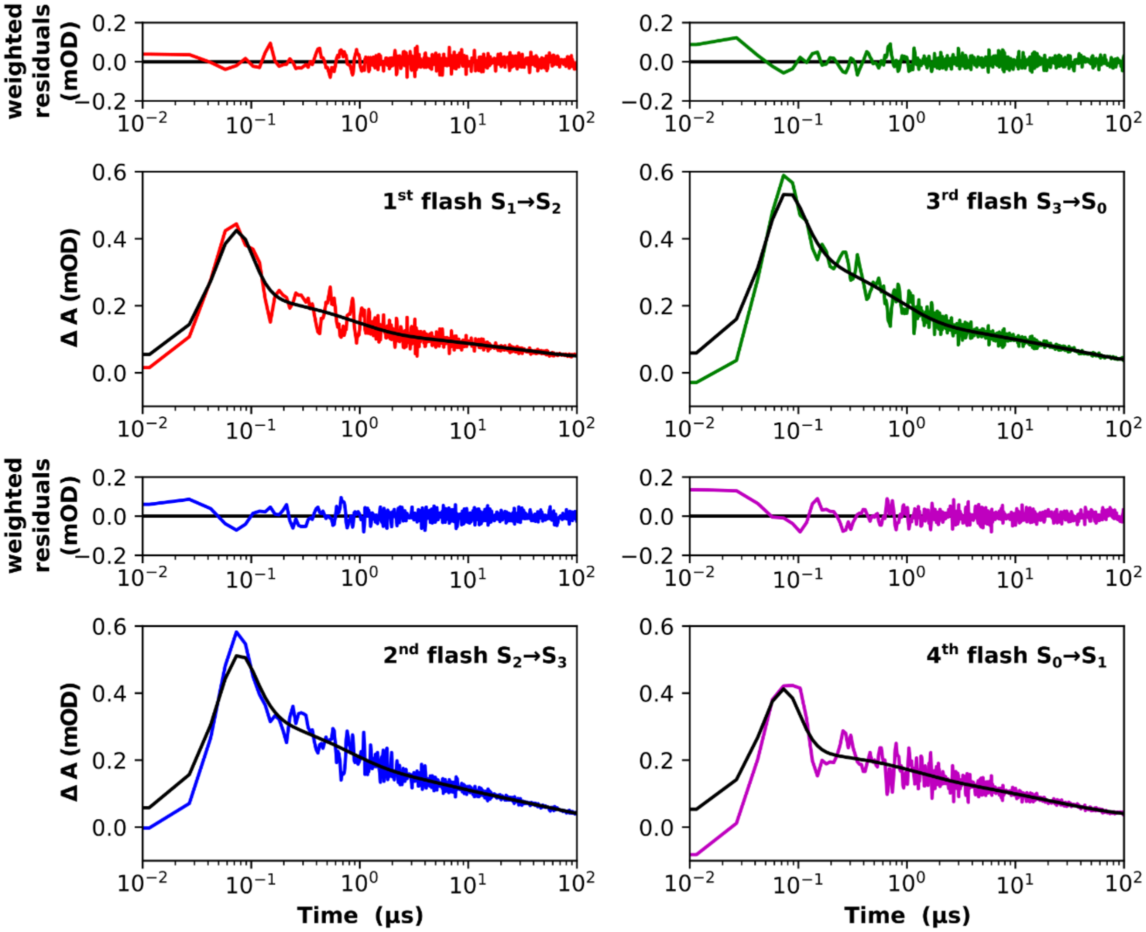

Figure S5: Multi-exponential fits of the P680$^+$ IR transients (averaged transients for wavenumbers greater than 1760 cm^{-1}), shown on a logarithmic x-axis. The colored lines indicate the recorded data while the black lines indicate the fits. Panels of lower height provide the deviation of the fits from the data, weighted by the averaged time per data point (weighted residuals). For each of the four transients, a four-component sum of exponentials plus offset (y_0) was fitted to the data. Data associated mostly with the $S_1 \rightarrow S_2$ transition (1st flash) is shown in red, the $S_2 \rightarrow S_3$ transition (2nd flash) in blue, the $S_3 \rightarrow S_0$ transition (3rd flash) in (green) and the $S_0 \rightarrow S_1$ (4th flash) in magenta. The shown data is identical to the data shown in Fig. 7 of the main paper.

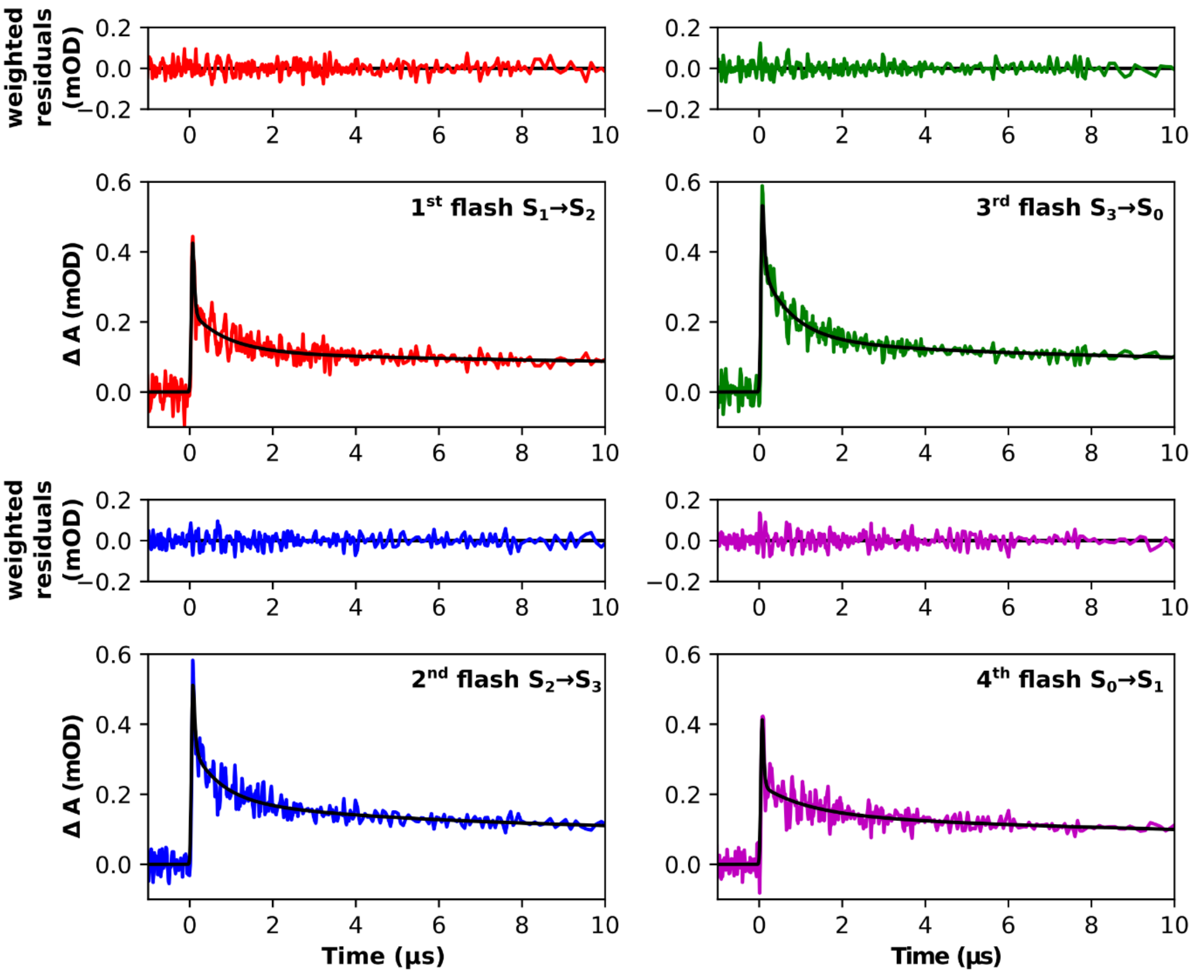

Figure S6: Multi-exponential fits of the P680⁺ IR transients (averaged transients for wavenumbers greater than 1760 cm^{-1}), of the first four excitation flashes on a linear time scale. The colored lines indicate the recorded data while the black lines indicate the fits. Panels of lower height provide the deviation of the fits from the data, weighted by the averaged time per data point (weighted residuals). For each of the four transients, a four-component sum of exponentials plus offset (y_0) was used as a fit function. Data associated mostly with the $S_1 \rightarrow S_2$ transition (1st flash) is shown in red, the $S_2 \rightarrow S_3$ transition (2nd flash) in blue, the $S_3 \rightarrow S_0$ transition (3rd flash) in (green) and the $S_0 \rightarrow S_1$ (4th flash) in magenta. The shown data is identical to the data shown in Fig. 7 and Fig. S5.

4. Additional Data: Experimental Artefact in the MHz Regime

In the early time domain of the time-resolved IR transients, we observe a recurring oscillatory behavior which we attribute to an experimental artefact (Fig. S7A). All flash-induced transients show oscillations that are approximately equal in phase, frequency and amplitude. Fig. S7C shows a Fourier transform of the averaged IR-transients of Fig. S7A, which clearly indicates the presence of an oscillatory feature of about 3.2 MHz. The origin of this artifact is still under investigation.

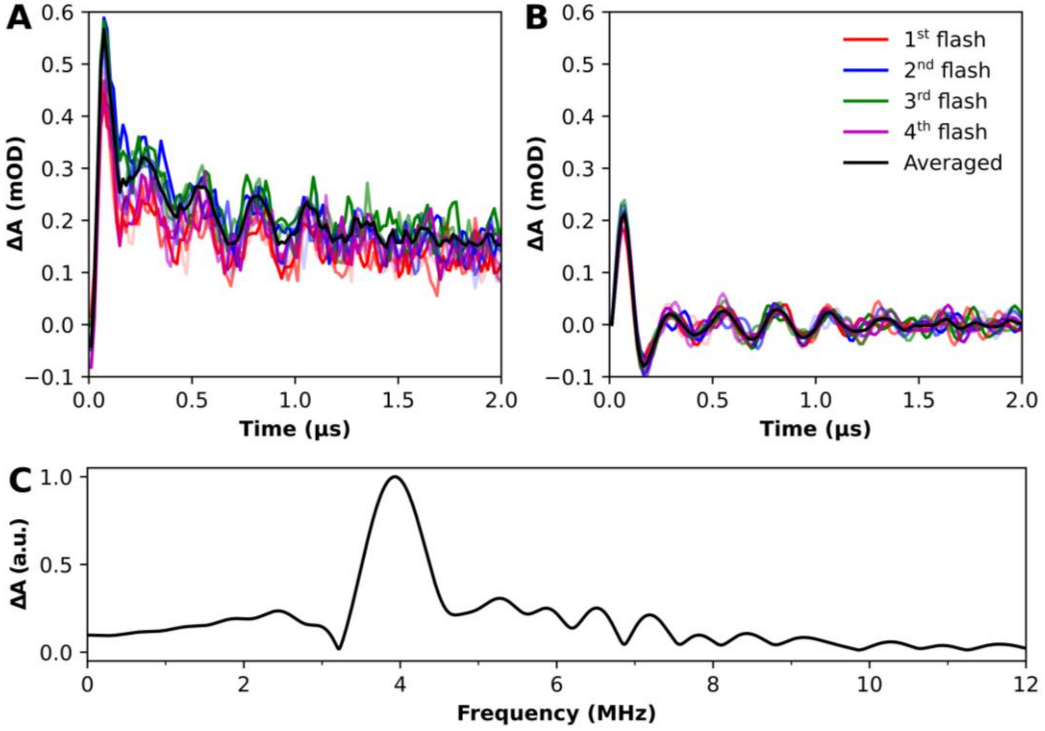

Figure S7: Experimental MHz artefact observed in the nanosecond to microsecond range of the IR transients. **(A)** Smoothed IR transients averaged for wavenumbers greater than 1760 cm^{-1}, for the first ten excitation flashes. The data, which had been logarithmically binned (averaged) during measurements, was interpolated to a linear time scale. All transients were shifted along the y-axis for better visual comparison, such that their value around 1.5 µs approaches zero. **(B)** A second order Buttwerworth bandpass filter was applied to the transients shown in (A) with limit frequencies of 1.8 MHz and 5 MHz. Clear oscillations can be seen in all recorded transients that extend well into the microsecond range. **(C)** Fourier transform of the data in (B), revealing that the oscillatory behavior has a frequency of around 4 MHz. The Fourier transform was performed after applying a 25% Tukey window and subsequent zero-filling to 4096 data points. The origin of this experimental artefact is still unclear. There are no indications that this artefact has affected the fit results reported in the present investigation significantly.

5. Additional Data: Acceptor-Side Kinetics

The band at 1478 cm^{-1} has previously been assigned to a C=0 mode of the Q_A^- semiquinone (Hienerwadel and Berthomieu 1995), making it a useful marker to observe acceptor side kinetics. An equivalent semiquinone mode of Q_B^- was observed to have its peak at 1480 cm^{-1} (Suzuki et al. 2005) and is thus also expected to contribute at 1478 cm^{-1}.

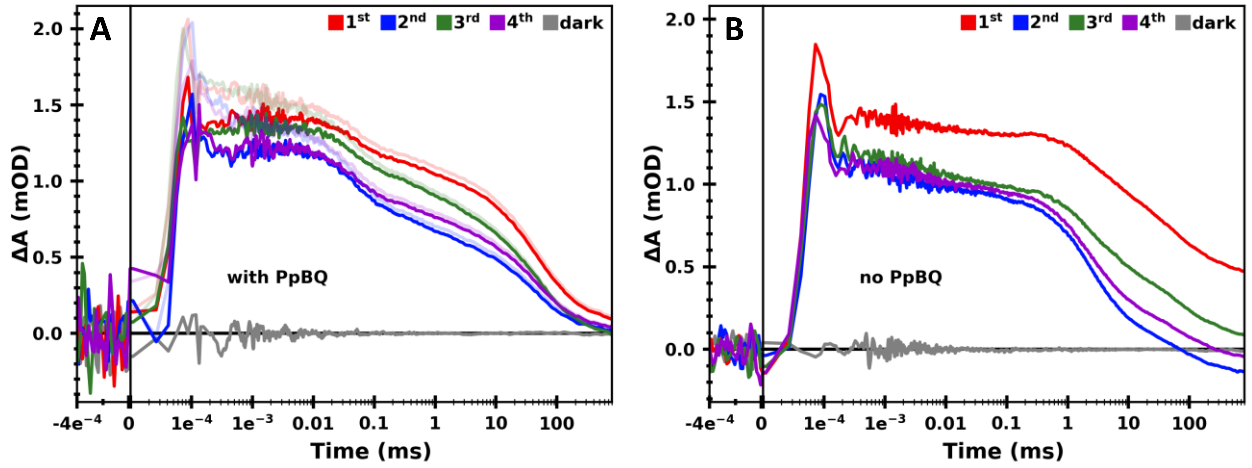

Fig. S8: Flash-induced difference absorption at 1478 cm^{-1}. **(A)** Transient absorption changes for the first four excitation flashes applied to dark-adapted PSII. The transients are shown with P680$^+$ background correction (opaque lines) and without (transparent lines) P680$^+$. The background-signal correction was done by subtraction of the averaged time course measured at wavenumbers greater than 1760 cm^{-1}. **(B)** Transient absorption changes of a PSII sample measured without the addition of exogenous electron acceptor PpBQ.

Acceptor Side Reactions in the Presence of PpBQ

Fig. S8(B) shows the absorption changes at 1478 cm^{-1} obtained *without* the addition of the artificial electron acceptor PpBQ. The signal does not decay to zero within 800 ms, but rather remains at a positive level after an odd number flashes and at a negative level after an even number of flashes. This can be explained by the generation of Q_B^- following an odd number of flashes, giving rise to a long-lived absorption at 1478 cm^{-1}. After an even number of flashes, Q_B^- is reduced to neutral Q_BH_2 and thus does not absorb at 1478 cm^{-1}; the difference absorption is thus lower than prior to the excitation flash.

In measurements with PpBQ (Fig. S8B) all transients decay to zero within 800 ms, with exception of the 1st flash transient. This is in line previous observations of PSII kinetics in the presence of PpBQ: Petrouleas and Diner (1987) found that applying an odd number of flashes does not lead to the formation of a semiquinone form of PpBQ in the Q_B binding site, but instead to the oxidation of the non-heme iron ($Fe^{2+} \rightarrow Fe^{3+}$) and thus to a double reduction of Q_B. Upon application of an even number of flashes, Q_B is not reduced at all, but instead the non-heme iron is reduced again. Thus, in the presence of PpBQ no long-lived semiquinones are expected, which is in agreement with the transients shown in Fig. S8(A).

The PpBQ chemistry affects also the kinetics detected at 1478 cm^{-1} (Fig. S8). Comparison of the Fig. S8A and Fig. S8B reveals a decay in the time range from 10 µs to 100 µs in the presence of PpBQ only; its amplitude is larger on even flash-numbers than on odd flash-numbers. This decay can be assigned to Q_A^- re-oxidation by electron transfer to the oxidized non-heme Fe (Fe^{3+}) formed in the relation to the above described PpBQ chemistry. This is predicted to occur in the here observed time range (Chernev et al. 2011). From these observations we can conclude that our PSII membrane particles do not lose their non-heme irons during the sample preparation procedure. A fraction of PSII is affected by the PpBQ chemistry involving Q_A^- reduction by the Fe^{3+} form of the non-heme Fe in PSII. This process of Q_A^- re-oxidation, however, does not affect the spectra of Fig. 2E to any significant extent because it becomes effective for times greater than 10 µs.

Contributions of Quinones to the Broad-band IR Absorption Changes

The transient absorption changes between 1760 – 1884 cm^{-1} decay in the early time range until reaching a plateau around 200 µs and then essentially remain stable until about 5 ms (see Fig. S4), followed by a decay within about 100 ms, which roughly matches the decay of the quinone signal detected at 1478 cm^{-1} (Fig. S9). If the absorption changes at those wavenumbers were purely due to $P680^+$, we would not expect a non-decaying signal component that is stable until about 5 ms, followed by a decay within about 100 ms. However, these are exactly the kinetic characteristics expected for a broad-band absorption increase assignable to quinone reduction. Thus we conclude that, most likely, in addition to the (dominant) broad-band absorption increase assignable to $P680^+$, there is broad-band absorption increase assignable to Q_A^- and Q_B^-. To quantify the contribution of the quinones to the broad background signal, the plateau region was averaged for the first four flash transients and divided by the initial signal (Fig. S10). We thereby estimate a quinone contribution of about 5.5 % in the 1st flash transient and about 3.5% in all following flash-induced transients. (The increase contribution on the first flash likely relates to a significant population of PSII where Q_A^- is stably accumulated, the so-called non-Q_B units.) In conclusion, both quinones of PSII, Q_A and Q_B, likely contribute to the here discussed broad absorption changes, but only to a very minor extent, in line with the data shown further below in Fig. S11.

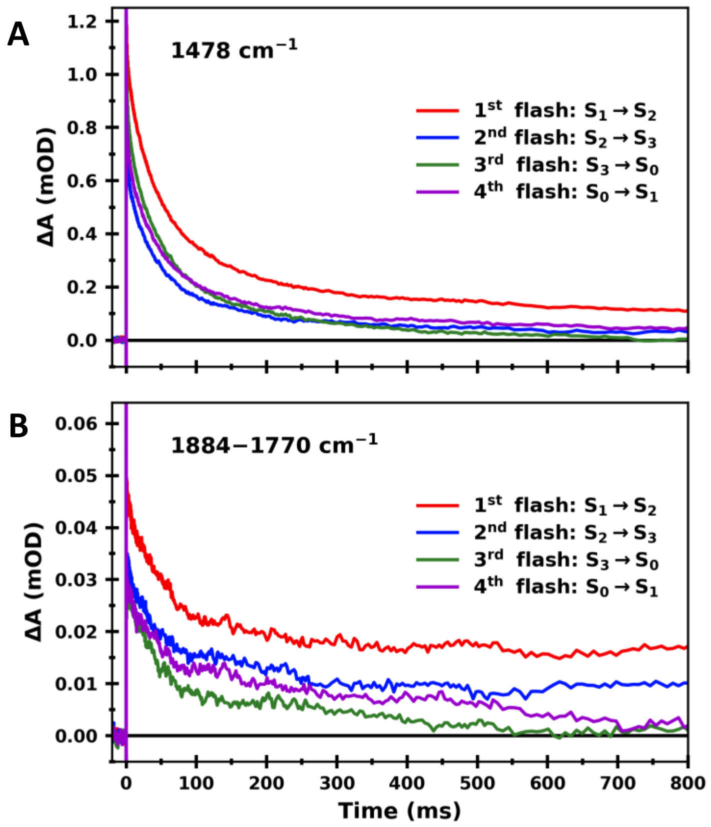

Fig. S9: Comparison of the millisecond kinetics at **(A)** 1478 cm^{-1} and **(B)** 1884-1760 cm^{-1}. The data is shown on a linear time scale; for easier visual inspection the fast sub-millisecond kinetics (i.e. the narrow spike around zero) was cropped. The semi-logarithmic display of the data in (A) and (B) is shown in Fig. S8A and Fig. S4, respectively.

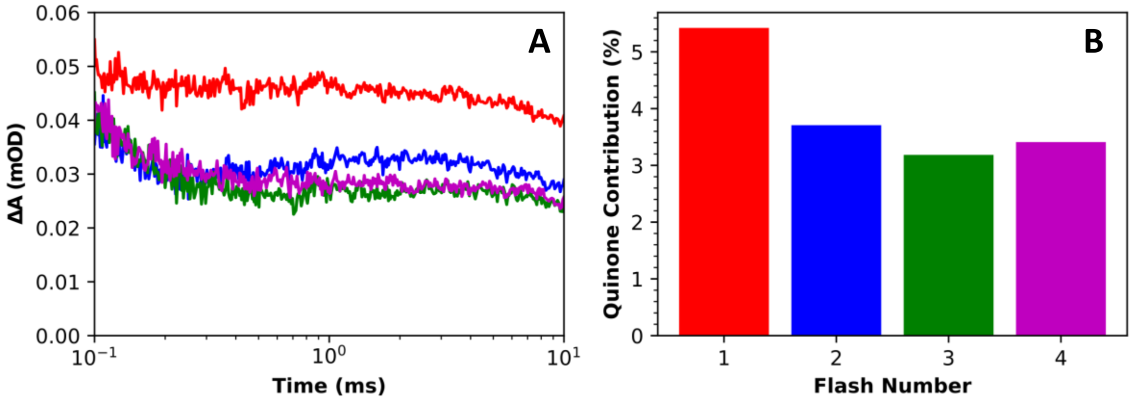

Fig. S10: Estimation of the quinone contribution to the broad background signal. **(A)** The averaged transients of the first four flashes between 1760 – 1884 cm^{-1} shown on a logarithmic x-axis between 0.1 and 10 ms. **(B)** Estimation of the quinone contribution of the broad background of the first four flash-induced transients, calculated by dividing the averaged plateau region (0.2 – 3 ms) by the initial peak. For the initial peak, the sum of all amplitudes plus y_0 of the fit results (see Tables S2 and S3) was used.

6. Additional Data: Flash-Number Dependence of Double-Difference Absorption

To address the question of whether we see quinone contributions in the double difference spectra of Fig. 2D/E of the main manuscript, we took a closer look at several wavenumbers that were previously associated with quinone bands, by Berthomieu et al. (1990) and Noguchi et al. (1999). The absence of a clear period-of-two flash-dependent behavior at most wavenumbers (Fig. S11) argues against a strong quinone contribution in the time range of 500 ns to 10 µs. The double difference flash-dependent behavior at 1532 cm^{-1} (Fig. S11D), however, may show a period-of-two oscillation (albeit weakly pronounced only). In conclusion, further careful studies will be necessary to truly access whether or not, and to what extent, acceptor-side kinetics contribute to the fast kinetics. (See also Discussion section of the article.)

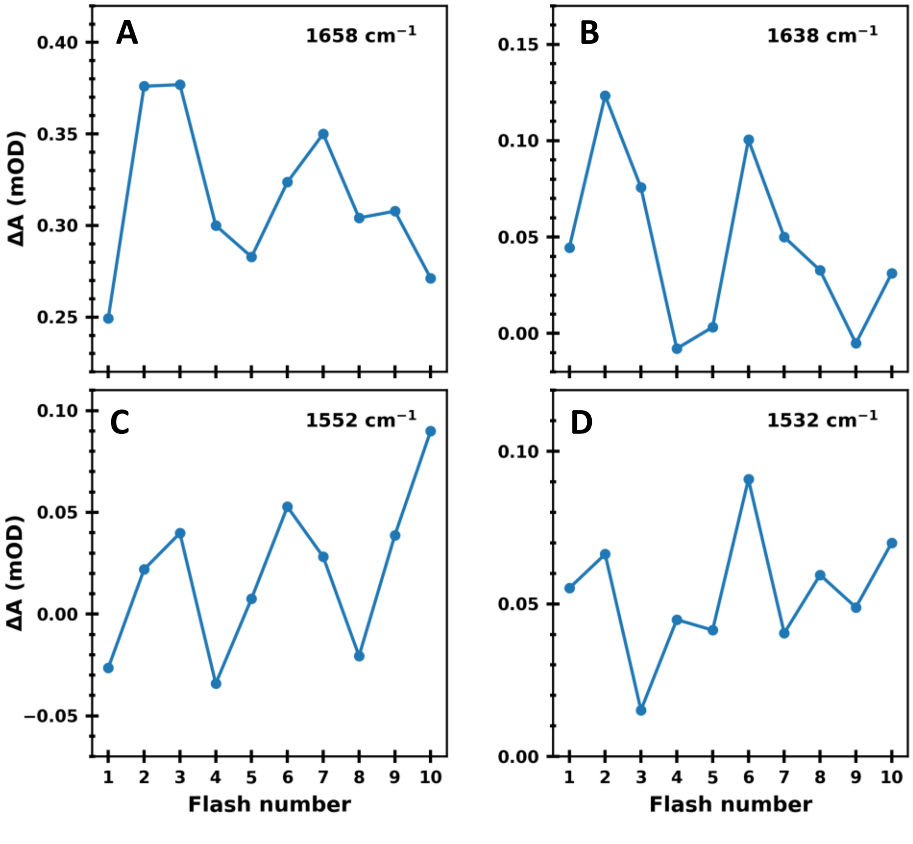

Fig. S11: Flash-number dependence of the IR double-difference absorption between 10 µs minus 500 ns at (A) 1658 cm^{-1}, (B) 1638 cm^{-1}, (C) 1552 cm^{-1}, (B) 1532 cm^{-1}. A sliding average over 3 neighboring wavenumbers was applied, as was done for the spectra shown in Fig. 2 of the main manuscript. The transients were corrected for the broad background signal, as in Fig. 2E.

References

Berthomieu, C, Nabedryk E, Mantele W, Breton J (1990) Characterization by FTIR spectroscopy of the photoreduction of the primary quinone acceptor Q_A in photosystem II. FEBS Lett. 269: 363-367. https://doi.org/10.1016/0014-5793(90)81194-S

Chernev, P, Zaharieva I, Dau H, Haumann M (2011) Carboxylate shifts steer inter-quinone electron transfer in photosynthesis. J Biol Chem 286: 5368-5374. https://doi.org/10.1074/jbc.M110.202879

Hienerwadel, R, Berthomieu C (1995) Bicarbonate binding to the non-heme iron of photosystem II, investigated by Fourier transform infrared difference spectroscopy and 13C-labeled bicarbonate. Biochemistry 34: 16288-16297. https://doi.org/10.1021/bi00050a008

Mäusle, SM, Agarwala N, Eichmann VG, Dau H, Nürnberg DJ, Hastings G (2023) Nanosecond time-resolved infrared spectroscopy for the study of electron transfer in photosystem I. Photosynth. Res. https://doi.org/10.1007/s11120-023-01035-9

Noguchi, T, Inoue Y, Tang X-S (1999) Hydrogen bonding interaction between the primary quinone acceptor Q_A and a histidine side chain in photosystem II as revealed by Fourier transform infrared spectroscopy. Biochemistry 38: 399-403. https://doi.org/10.1021/bi982294v

Petrouleas, V, Diner BA (1987) Light-induced oxidation of the acceptor-side Fe(II) of Photosystem II by exogenous quinones acting through the QB binding site. I. Quinones, kinetics and pH-dependence. Biochim. Biophys. Acta 893: 126-137. https://doi.org/10.1016/0005-2728(87)90032-6

Suzuki, H, Nagasaka M-a, Sugiura M, Noguchi T (2005) Fourier transform infrared spectrum of the secondary quinone electron acceptor Q_B in photosystem II. Biochemistry 44: 11323-11328. https://doi.org/10.1021/bi051237g
